# β-catenin mediates endodermal commitment of human ES cells via distinct transactivation functions

**DOI:** 10.1186/s13578-024-01279-5

**Published:** 2024-07-24

**Authors:** Xun Ma, Liujiang Dai, Chunlai Tan, Jiangchuan Li, Xiangjun He, Yaofeng Wang, Junyi Xue, Min Huang, Jianwei Ren, Yin Xia, Qiang Wu, Hui Zhao, Wai-Yee Chan, Bo Feng

**Affiliations:** 1 School of Biomedical Sciences, Faculty of Medicine, CUHK-GIBH CAS Joint Research Laboratory on Stem Cell and Regenerative Medicine, The Chinese University of Hong Kong, Hong Kong SAR, China; 2 Centre for Regenerative Medicine and Health, Hong Kong Institute of Science & Innovation, Chinese Academy of Sciences, Hong Kong SAR, China; 3 The State Key Laboratory of Quality Research in Chinese Medicine, Macau University of Science and Technology, Macau SAR, China; 4 Guangzhou Institute of Biomedicine and Health, Chinese Academy of Sciences, Guangzhou, 510530 China; 5 The Chinese University of Hong Kong, Shenzhen Research Institute, Shenzhen, 518000 China

## Abstract

**Background:**

β-catenin, acting as the core effector of canonical Wnt signaling pathway, plays a pivotal role in controlling lineage commitment and the formation of definitive endoderm (DE) during early embryonic development. Despite extensive studies using various animal and cell models, the β-catenin-centered regulatory mechanisms underlying DE formation remain incompletely understood, partly due to the rapid and complex cell fate transitions during early differentiation.

**Results:**

In this study, we generated new *CTNNB1-/-* human ES cells (hESCs) using CRISPR-based insertional gene disruption approach and systematically rescued the DE defect in these cells by introducing various truncated or mutant forms of β-catenin. Our analysis showed that a truncated β-catenin lacking both N- and C-terminal domains (ΔN^148^C) could robustly rescue the DE formation, whereas hyperactive β-catenin mutants with S33Y mutation or N-terminal deletion (ΔN^90^) had limited ability to induce DE lineage. Notably, the ΔN^148^C mutant exhibited significant nuclear translocation that was positively correlated with successful DE rescue. Transcriptomic analysis further uncovered that two weak β-catenin mutants lacking the C-terminal transactivation domain (CTD) activated primitive streak (PS) genes, whereas the hyperactive β-catenin mutants activated mesoderm genes.

**Conclusion:**

Our study uncovered an unconventional regulatory function of β-catenin through weak transactivation, indicating that the levels of β-catenin activity determine the lineage bifurcation from mesendoderm into endoderm and mesoderm.

**Supplementary Information:**

The online version contains supplementary material available at 10.1186/s13578-024-01279-5.

## Introduction

The Wnt/β-catenin signaling pathway plays a pivotal role during early embryonic development. β-catenin, as the core effector of this pathway, is directly involved in the process of forming definitive endoderm (DE), a prevenient lineage that subsequently gives rise to all the endodermal tissues and organs [1, 2]. However, the cellular mechanisms underlying β-catenin's function in this process remain incompletely understood due to its complex functionality. On one hand, canonical Wnt signal triggers nuclear translocation of β-catenin to drive transcriptional programs through cooperation with other nuclear transcription factors such as TCF/LEF [3, 4]. On the other hand, β-catenin also serves as a component of adherens junctions, interacting with E-cadherins and α-catenin/F-actin to maintain cell–cell contacts and modulate cell migration [5–8]. Hence, it has been technically challenging to delineate the multifaceted cellular roles of β-catenin during the rapid cell fate transitions in embryos.

Embryonic stem cells (ESCs) derived from mouse and human blastocysts retain the pluripotency property of early embryos and can differentiate into DE lineage in culture, in the presence of Activin and Wnt [9–11]. This process resembles in vivo DE formation and provides valuable models to delineate underlying mechanisms in vitro [12]. Mouse ESCs (mESCs) with a knockout of β-catenin *Ctnnb1-/-*) have been generated through multiple targeting strategies [13–16]. Despite normal proliferation and stable expression of pluripotency markers in the undifferentiated stage, these β-catenin knockout mESCs consistently failed to differentiate into mesodermal and endodermal lineages. Recently, human ESCs (hESCs) carrying β-catenin knockout (*CTNNB1-/-*) were generated through CRISPR-introduced mutagenesis [17, 18]. Similarly, these cells retained normal hESC properties under undifferentiated conditions but failed to form embryoid bodies [18] and were unable to differentiate into mesoderm upon induction [17]. Although β-catenin has been widely demonstrated to be required for DE differentiation using various in vivo and in vitro models, further investigations in mESCs found that β-catenin may not regulate DE commitment through its conventional TCF-dependent transactivation activity. Lyashenko et al. showed that truncated β-catenin lacking nuclear function for TCF/LEF-based transactivation could still induce DE differentiation from *Ctnnb1-/-* mESCs [15]. Moreover, a recent study in HEK293T cells also reported the finding of β-catenin mediated transactivation independent of TCFs [19]. These findings suggested that β-catenin may possess an undisclosed unconventional activity involved in controlling DE differentiation.

In this study, we generated new *CTNNB1*-/- hESCs through a homology-independent insertional gene-disruption approach [20, 21] and confirmed that the *CTNNB1* null hESC clones were unable to differentiate into DE lineage. By introducing various truncated or mutant forms of β-catenin using lentivirus, we found that DE differentiation defect in *CTNNB1*-/- hESCs could be effectively rescued not only by the full-length (FL) β-catenin but also by several mutants with distinct deletions. We verified the transactivation functions of truncated β-catenin mutants and identified interesting correlations between their nuclear activities and the rescue outcomes. RNA-seq analysis was also performed to probe the transcriptional responses triggered by different β-catenin mutants, which further supported that an unconventional CTD-independent transcription regulatory function of β-catenin played an important role to control the DE commitment from hESC.

## Methods

### Constructs

#### Lenti-CTNNB1 / CTNNB1-mutants / CDH1

The full-length coding sequences (CDS) of human *CTNNB1* transcript variant 1 (NM_001904.3) (2346 bp) and *CDH1* transcript variant 1 (NM_004360.5) (2649 bp) were amplified from H1 hESCs by RT-PCR and cloned into Fuw-tetO lentiviral vector carrying Dox-inducible CMV promoter (Addgene #20726) [22]. Truncated and mutant *CTNNB1* CDS encoding various β-catenin mutants were then generated by PCR amplification from the FL *CTNNB1* cDNA and sub-cloned into the same Fuw-tetO vector. For ΔN^148^C and ΔC mutants, 3 × Flag-tag (DYKDDDDK) coding sequences were inserted at the 3’-end of the truncated *CTNNB1* cDNA, to generate fusion proteins for immunostaining detection.

#### Luciferase reporters

TOPFlash luciferase reporter (Fig. 2B) was modified from TOP-GFP plasmid (Addgene #35489) by replacing GFP with the Firefly luciferase gene. Luciferase reporters for enhancer analysis (Figs. 4F and 5E) were constructed using pGL4.23[luc2/minP] vector (Promega). Individual enhancer elements from *TBXT, EOMES, MIXL1,* and *NODAL* gene locus were PCR amplified from H1 hESC genome DNA and inserted upstream of the mini promoter at BglII site.

*Cas9 and sgRNAs:* Plasmid encoding human codon optimized spCas9 (Addgene #41815) was a gift from George Church [23]. The sgRNA backbone vector was modified from MLM3636 (Addgene #43860), a gift from Keith Joung, as previously described [20]. sgRNAs were designed using “CRISPR Design” developed by Feng Zhang’s laboratory (http://crispr.mit.edu/) and constructed as described previously [23]. Activities of sgRNAs were examined by T7E1 assay after transfection in HEK293T cells [20] to identify the best performer. In this study, sgCTNNB1 targeting 5’-TCCCAAGTCCTGTATGAGT-3’ and sgCDH1 targeting 5’-GGACGCGGCCTCTCTCCAGG-3’ were used to knockout *CTNNB1* and *CDH1* gene, respectively.

#### NHEJ donors

The NHEJ donor plasmid carrying ires-eGFP (Addgene #83575) and ires-TdTomato were generated in our previous studies [20, 21].

### Cell culture and DE differentiation of hESCs

H1 human ESCs (WiCell Research Institute) were cultured as previously described [20] on Matrigel (BD Biosciences) coated plates under feeder-free conditions in mTeSR1 medium (STEMCELL Technologies). Medium was refreshed daily, and cells were subcultured every 3–4 days at a 1:3 split ratio. DE differentiation was induced by adopting previously described methods [9–11]. Briefly, wt hESCs (H1) were seeded one day before to reach about 25% confluence. The mTeSR1 medium was then replaced by RPMI 1640 supplemented with 1 × B27 supplement (Gibco) and 100 ng/ml Activin A (R&D Systems, Minneapolis, MN), and refreshed every day. For wt hESCs (H1), 3 μM CHIR99021 (CHIR) (Cayman) was added for the first day of the DE induction. For the *CTNNB1-/-* hESCs or rescue clones carrying different β-catenin mutants, 0.5 μg/ml Doxycycline (Dox) (Sigma) was added at one day before the DE induction and removed at day 2 post induction.

HEK293T cells (ATCC) were cultured in Dulbecco's modified Eagle's medium (DMEM) supplemented with 10% fetal bovine serum (FBS) (Thermo Fisher Scientific). All cells were incubated at 37 °C and 5% CO_2_.

### Generation of *CTNNB1-/-* hESCs, *CDH1-/-* hESCs and *CTNNB1-/-* HEK293T cells via CRISPR-based insertional gene disruption

The CRISPR-mediated NHEJ-based insertional disruption of *CTNNB1* or *CDH1* gene in H1 hESCs and in HEK293T cells were performed as described previously [20, 21]. Briefly, the plasmids encoding ires-GFP/TdTomato (donor), spCas9 and relevant sgRNAs were co-transfected into H1 hESCs or HEK293T cells. At day 5 to day 7 post transfection, the transfected cells were analyzed and GFP + (or TdTomato +) cells were collected through FACS using BD FACSAria™ II system (BD Biosciences). These genome-edited cells were then seeded at low density (< 1000 cells per 6-well) and single clones were expanded. Genome PCRs were performed to identify the correct clones carrying biallelic disruption of target genes, through either donor insertion or frameshifting indels. The primers used were listed in Table S1.

### Lentivirus preparation, transduction, and single-cell clone expansion

The lentiviruses were generated as previously described [22]. Briefly, HEK293T cells were co-transfected with pMD2.G (Addgene #12259), psPAX2 (Addgene #12260), and a specific lentiviral vector constructed above. The undifferentiated *CTNNB1-/-* hESCs and specific rescue clones were transduced with corresponding lentiviruses in mTESR1 medium supplemented with 4 μg/ml polybrene (Sigma-Aldrich). The transduced cells were maintained for 3–5 days and dissociated into single cells using TrypLE (Gibco). These single cells were then seeded at low density (< 1000 cells/ 6-well) in mTeSR1 supplemented with 10 µM Y-27632 (STEMCELL Technologies) to raise single-cell clones. Dox-treatment followed by qRT-PCR analysis were performed to identify the correct clones carrying Dox-induced expression of specific *CTNNB1* mutants or *CDH1*.

(Other methods were provided in the Supplementary Information).

## Results

### Analysis using new *CTNNB1-/-* hESCs demonstrated that β-catenin is essential to DE induction

**Fig. 1:**
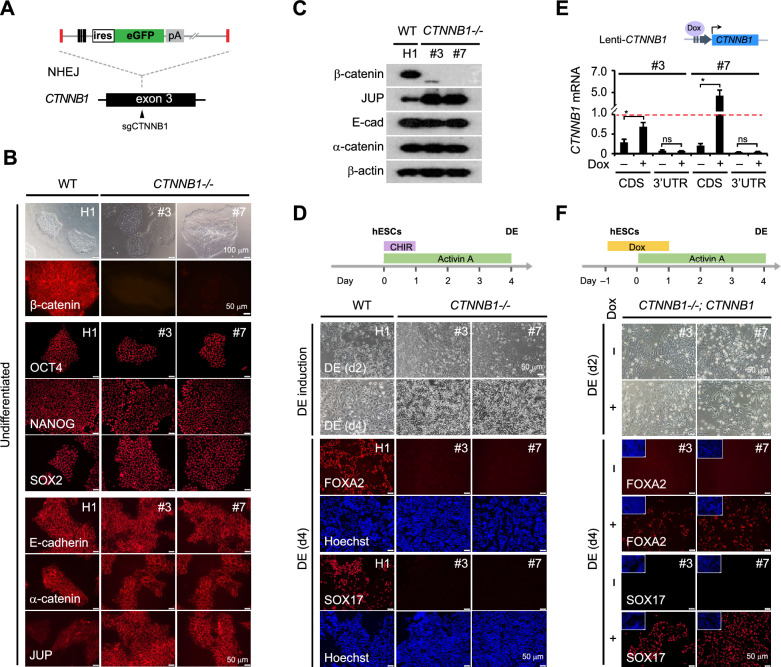
Generation of new *CTNNB1-/-* hESC clones and rescue of DE differentiation by ectopic expression of β-catenin. **A** Schematics of the donor and CRISPR/Cas9-based targeting strategy for insertional disruption of *CTNNB1* gene via NHEJ mechanism. **B** Morphology and immunostaining of undifferentiated wt hESC (H1) and *CTNNB1-/-* clones (#3 and #7). Bright field images were shown with scale bar 100 μm. Immunostainings were performed using antibodies specific to β-catenin, pluripotency marker OCT4, NANOG, SOX2, and membrane protein E-cadherin, α-catenin and JUP. Scale bars = 50 μm in fluorescence images. **C** Western blot detection of β-catenin, JUP, E-cadherin, and β-catenin proteins in undifferentiated wt hESC (H1) and *CTNNB1-/-* clones (#3 and #7). **D** DE induction from wt hESC (H1) and *CTNNB1-/-* clones (#3 and #7). Shown are the workflow diagram for DE induction (upper) and results of DE differentiation (lower). Bright field images were taken at day 2 (d2) and day 4 (d4) post DE induction. Immunostaining for DE marker FOXA2 and SOX17 were performed at DE (d4). Nuclei were counterstained using Hoechst. Scale bars = 50 μm. **E** Lentivirus-delivered *CTNNB1* expression in *CTNNB1-/-* clones. Shown are schematics of the Dox-inducible *CTNNB1* cassette delivered by lentivirus (Lenti-*CTNNB1*) (upper) and qRT-PCR results from the transduced *CTNNB1-/-* clones (#3 and #7) (lower), which confirmed the Dox-induced expression of *CTNNB1* transgene (CDS) and constant depletion of endogenous *CTNNB1* mRNA (3’UTR). Data were normalized to wt hESC (H1) (red dashed line) and presented as mean ± SD. Student’s t-test was performed between Dox-induced and non-induced cells. *, *p* ≤ 0.05. **F** DE induction from Lenti-*CTNNB1* transduced *CTNNB1-/-* clones (#3 and #7). Shown are the workflow diagram for Dox-based DE induction (upper) and results of DE differentiation in the absence (–) and presence (+) of Dox (lower). Bright-field images were taken at DE (d2) and immunostainings were performed for FOXA2 and SOX17 at DE (d4). Nuclei were counterstained using Hoechst (shown in small box). Scale bars = 50 μm

To investigate how β-catenin is exactly involved in controlling DE differentiation, we generated new types of *CTNNB1-/-* hESCs using CRISPR-based insertional disruption approach through non-homologous end joining (NHEJ) mechanism [20, 21]. The donor carrying ires-GFP was inserted at *CTNNB1* exon-3 to disrupt endogenous *CTNNB1* gene (Fig. 1A). Based on GFP expression (Fig. S1A), we identified two clones (named #3 and #7) that carried biallelic disruption of *CTNNB1* gene (Fig. S1B, upper) and were unable to produce full-length *CTNNB1* mRNA (Fig. S1B, lower) and β-catenin protein (Fig. S1C, D). Both #3 and #7 clones maintained normal hESC morphology, stable expression of hESC markers (OCT4, NANOG and SOX2), and unchanged localization of E-cadherin and α-catenin at cell junctions (Fig. 1B). As a compensatory response to the loss of β-catenin [15, 18], higher levels of JUP were detected in these *CTNNB1-/-* clones (#3 and #7) than in wt hESCs (H1) (Fig. 1B, C), supplementing the maintenance of stable cell–cell adhesion and normal colony morphology.

We found that both #3 and #7 *CTNNB1-/-* hESCs failed in the DE differentiation induced using Activin A and CHIR99021 (Fig. 1D). These cells didn’t adopt the epithelial-mesenchymal transition (EMT) which was consistently observed in wt hESCs (H1) at day 2 post DE induction [24], and they were unable to activate DE markers *SOX17* and *FOXA2* at day 4 after induction (Fig. 1D). Importantly, ectopic expression of full-length (FL) β-catenin, which were delivered via lentivirus transduction and induced with doxycycline (Dox), rescued the DE differentiation of both KO#3 and KO#7 clones. EMT-like morphology was significantly restored at day 2 and robust activation of *SOX17* and *FOXA2* was observed at day 4 after DE induction (Fig. 1E, F). These results collectively demonstrated that β-catenin is an essential player in mediating the DE differentiation from hESCs.

### Different β-catenin functional mutants yielded varied DE rescue outcomes

**Fig. 2:**
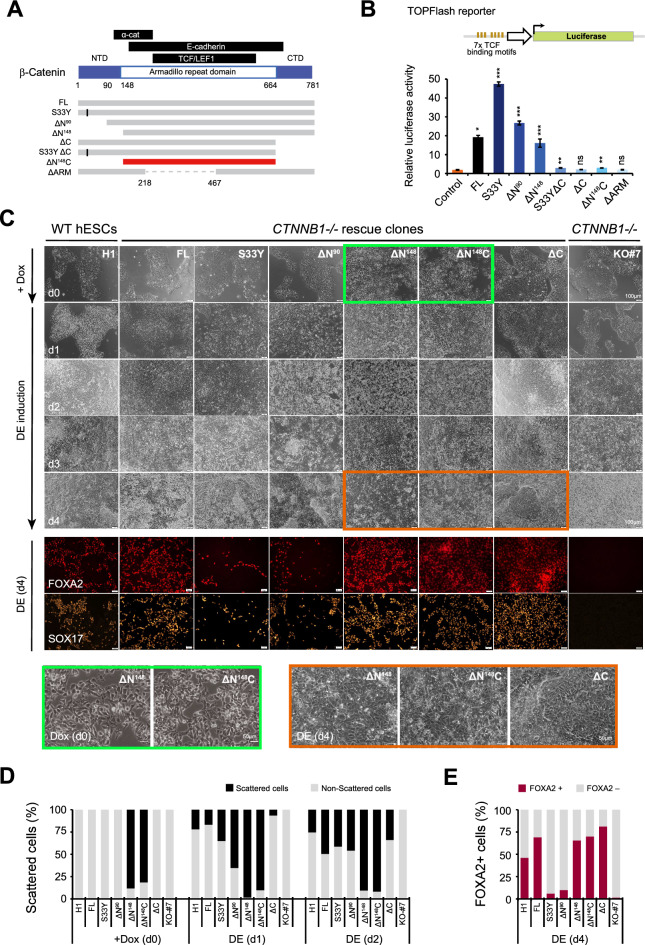

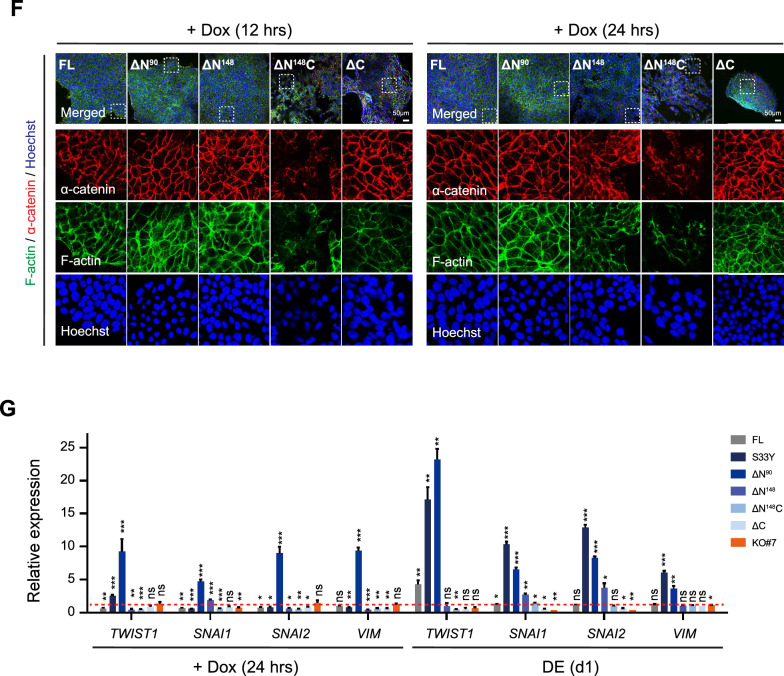
Diverse rescue of DE differentiation by different β-catenin mutants. **A** Schematics of β-catenin protein and various engineered mutants. Different functional domains were indicated. Positions shown were amino acid residues. **B** TOPFlash reporter assays. Shown were the schematics of reporter construct (upper) and the luciferase activities reflecting TCF-dependent transactivation induced by FL β-catenin and various mutants (lower). The assays were performed using *CTNNB1-/-* HEK293T cells (Fig. S2). Data shown are mean ± SD. Student’s t-test was performed between individual test and untransfected control cells. ns, not significant; *, *p* ≤ 0.05; **, *p* ≤ 0.01; ***, *p* ≤ 0.001. **C** DE induction processes from wt hESC (H1), *CTNNB1-/-* clone (KO#7), and single-cell clones rescued with FL β-catenin and different mutants in *CTNNB1-/-* hESCs (KO#7) background. Brightfield images were taken every day from d0 to d4 during DE induction. Scale bars = 100 μm. Close views of the bright field images in green and orange boxes were shown below (scale bars = 50 μm). Immunostaining of FOXA2 and SOX17 were performed at DE (d4). Nuclei were counterstained using Hoechst. Scale bars = 50 μm. **D** Percentage of scattered cells observed at d0, d1 and d2 during DE induction from the cell clones examined in **C**. The calculation was performed using Image J. **E** Ratio of FOXA2-positive cells at DE (d4) from the cell clones examined in **C**. The calculation was performed using Image J. **F** Immunostaining of α-catenin (red) and F-actin (green) in different rescue clones, at 12 h and 24 h after Dox treatment. Nuclei were counterstained using Hoechst (blue). Shown were images with merged signals (upper) and close views of selected areas (indicated by boxes) presented in individual channels. Scale bars = 50 μm. **G** Relative expression (fold) of EMT marker genes (*TWIST1, TWIST2, SNAI1, SNAI2* and *VIM*) after Dox treatment for 24 h or at DE (d1). The qRT-PCR data in *CTNNB1-/-* hESC (KO#7) and various rescue clones were normalized to that in undifferentiated wt hESCs (H1) (red dashed line) and presented as mean ± SD (n = 3). For statistical analysis, all data were compared to that of undifferentiated wt hESCs (H1). ns, not significant; *, *p* ≤ 0.05; **, *p* ≤ 0.01; ***, *p* ≤ 0.001

Next, to examine which functional domain of β-catenin is crucial for DE differentiation, we generated multiple β-catenin mutants, each carrying a distinctive deletion/mutation to ablate specific functional domain(s) (Fig. 2A). Transactivation activities of these β-catenin mutants were examined through TOPFlash assay, in which, *CTNNB1-/-* HEK293T cells generated with the CRISPR strategy were used to avoid the interference by endogenous β-catenin (Fig. 2B and Fig. S2). Our results showed that the β-catenin mutants carrying S33Y mutation or N-terminal deletion of 1–90 aa (ΔN^90^), which were both resistant to cytoplasmic degradation [25, 26], indeed yielded drastically enhanced TOPFlash activities compared to FL β-catenin (Fig. 2B). The ΔN^148^ mutant harboring a longer N-terminal deletion (1–148 aa) including α-catenin interaction motif (118–146 aa) also produced substantial TOPFlash signal (Fig. 2B). In contrast, deletion of C-terminal transactivation domain (CTD) (665–781 aa) (ΔC, S33YΔC, and ΔN^148^C mutants) or partial removal of central armadillo repeat (ARM) region (218-467aa) (ΔARM mutant) largely abolished the TCF-dependent transactivation of TOPFlash reporter (Fig. 2B).

We then introduced these β-catenin mutants with Dox-inducible expression into the #7 *CTNNB1-/-* hESC (KO#7) via lentivirus transduction. The herein generated single-cell clones all maintained normal hESC morphology and expressed pluripotency markers in the absence of Dox (Fig. S3), while expressing specific β-catenin mutants upon Dox induction (Fig. S4). DE differentiations of these clonal cells were then examined, and Dox was supplemented to induce the expression of β-catenin or mutants to mimic Wnt activation. Interestingly, the defects of DE differentiation in the *CTNNB1-/-* hESCs were successfully rescued by multiple β-catenin mutants, whereas the morphological changes and levels of DE marker activation were greatly varied (Fig. 2C–E). The ΔN^148^ and ΔN^148^C clones underwent rapid cell scattering upon Dox treatment (d0) and colonies dispersed even before Activin A was supplemented for DE induction (Fig. 2C, green box; Fig. 2D), while the FL, S33Y, ΔN^90^ and ΔC clones maintained intact colonies resembling wt H1 hESCs under the same condition (Fig. 2C, D). At day 1 post DE induction (d1), dramatic cell scattering was also observed in S33Y and ΔN^90^ clones, while wt hESC (H1) and FL β-catenin rescue clone only showed minor morphological changes (Fig. 2C, D). In the ΔC clones, the cell scattering phenotype was further delayed and became evident only at day 2 post DE induction (d2) (Fig. 2C). Interestingly, after 4 days of induction (d4), FL, ΔN^148^, ΔN^148^C and ΔC clones all gave rise to epithelial-like cells resembling the DE phenotype (Fig. 2C, reddish box), while the S33Y and ΔN90 clones largely produced fibroblasts-like cells (Fig. 2C). Consistently, immunostaining detected high proportions of DE cells expressing FOXA2 and SOX17 among the FL, ΔN^148^, ΔN^148^C and ΔC clones at DE (d4), whereas ΔN^90^ and S33Y clones yielded much fewer positive cells (Fig. 2C, E). These results showed that the truncation mutants ΔN^148^, ΔN^148^C and ΔC could rescue β-catenin function in mediating DE differentiation, while surprisingly, the dominant and hyperactive mutant S33Y and ΔN^90^ yielded the least rescue effects. The lack of correlation between the DE rescue outcomes (Fig. 2C–E) and TOPFlash activities (Fig. 2B) among different β-catenin mutants suggested that β-catenin might have CTD-independent functions which played important roles in DE differentiation.

### Truncated β-catenin ΔN^148^ and ΔN^148^C disrupted cell–cell adhesion without inducing EMT process

To clarify if the cell-scattering phenotype induced by ΔN^148^ and ΔN^148^C promoted the EMT process associated with DE differentiation [24], we further examined the timing of cell junction breakdown and EMT gene activation during DE induction. Markedly, at as early as 12 h (hrs) post Dox treatment, dramatic decrease and dislocation of α-catenin and F-actin from cell junctions were observed in ΔN^148^C clone (Fig. 2F, left panel), which was well correlated with its early cell scattering phenotype (Fig. 2C, D). Similar morphological changes were observed later in ΔN^148^ clone at 24 h post Dox treatment, but not in other rescue clones examined at the same time (Fig. 2F, right panel). To further address if an active EMT progress was induced or even enhanced during the cell scattering, we examined the expression of EMT genes. Interestingly, no evident activation of EMT markers was detected in ΔN^148^ and ΔN^148^C clones, either after Dox-treatment or at DE (d1) (Fig. 2G). Whereas *TWIST1, SNAI1/2, and VIM* were dramatically activated in S33Y and ΔN^90^ clones and moderately induced in the FL and ΔN^148^ cells at DE (d1) (Fig. 2G), supporting that EMT was induced. These results indicated that the cell scattering phenotype observed in ΔN^148^ and ΔN^148^C clones was not associated with EMT activation. Instead, due to the lack of α-catenin interaction motif, ΔN^148^ and ΔN^148^C were unable to maintain adherens complex in cell junctions and became dominant-negative (DN) mutants. Hence, cell–cell junctions that were maintained by elevated JUP in the absence of β-catenin were disrupted (Fig. 1B, C), causing cell scattering. This notion undermined the involvement of β-catenin membrane functions in DE commitment.

### Truncated ΔN^148^C β-catenin showed nuclear translocation correlated with DE rescue

**Fig. 3:**
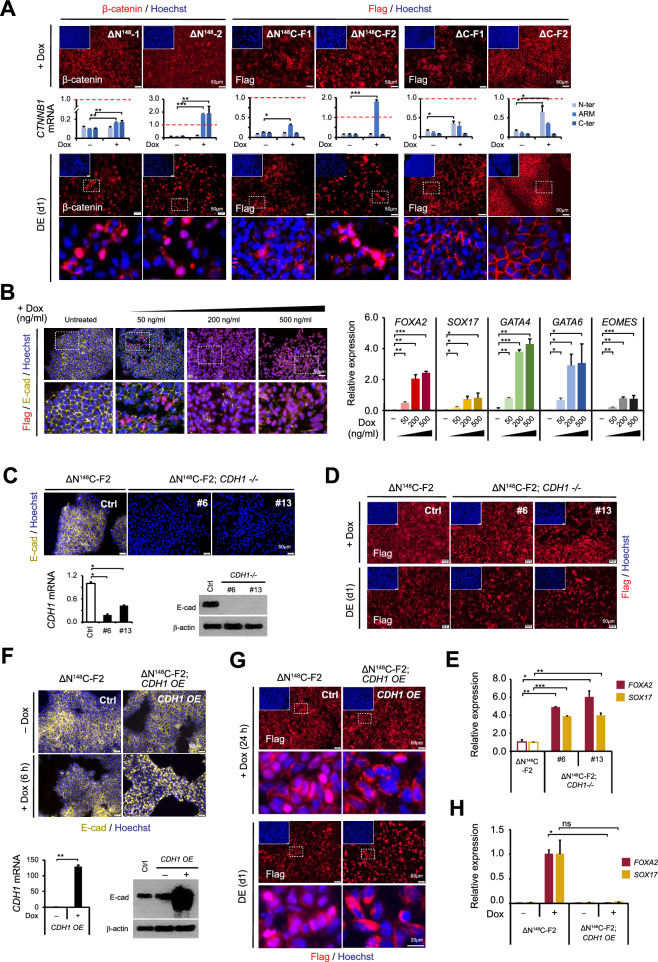
Dose-dependent nuclear translocation and E-cadherin sequestration of ΔN^148^C mutant affect DE rescue outcomes. **A** Nuclear translocation of ΔN^148^ and ΔN^148^C β-catenin mutants. Immunostaining of the ΔN^148^, ΔN^148^C and ΔC clones were performed after Dox treatment for 24 h (upper panels) and at DE (d1) (lower panels). Antibodies used were specific to β-catenin or Flag-tag. Two individual rescue clones were analyzed for each β-catenin mutant. The Dox-induced expression of *CTNNB1* mutant transgenes were verified by qRT-PCR (middle panel) using primers binding to N-terminal (N-ter), central (ARM), and C-terminal (C-ter) regions in the *CTNNB1* CDS. The data values were normalized to wt hESCs (H1) (dashed red lines). **B** Dose-dependent nuclear translocation of ΔN^148^C (left) and corresponding DE differentiation (right). Left panels showed the immunostaining of ΔN^148^C-F2 clone cells treated with Dox at different concentrations (indicated) for 24 h. Antibodies used were specific to E-cadherin (yellow) and Flag-tag (red). Shown were images with merged signals and close views of selected areas inside the dashed line boxes were shown below. Right panel showed the qRT-PCR analysis of DE-related genes (*FOXA2, SOX17, GATA4, GATA6* and *EOMES*) at day 4 post DE induction, using Dox at different concentrations. The data values were normalized to that in wt hESCs (H1). For statistical analysis, all data were compared to that of non-induced cells. **C** E-cadherin expression in *CDH1-/-* hESCs derived from ΔN^148^C-F2 clone. Immunostaining of ΔN^148^C-F2 clone and ΔN^148^C-F2;*CDH1-/-* clones (#6 and #13) were performed. Antibody used were specific to E-cadherin (yellow). qRT-PCR and western blot quantified *CDH1* mRNA and E-cadherin protein levels, respectively (lower panels). **D** Nuclear translation of ΔN^148^C in *CDH1-/-* hESC. Immunostaining of ΔN^148^C-F2 clone and ΔN^148^C-F2;*CDH1-/-* clones (#6 and #13) were performed after Dox treatment for 24 h (upper panels) and at DE (d1) (lower panels). Antibodies used were specific to Flag-tag (red). **E** Relative expression (fold) of *FOXA2* and *SOX17* at DE (d4) in ΔN^148^C-F2 clone and ΔN^148^C-F2;*CDH1-/-* clones (#6 and #13). For statistical analysis, data of ΔN^148^C-F2;*CDH1-/-* clones were compared to that of ΔN^148^C-F2 cells. **F** E-cadherin levels in *CDH1-*transduced ΔN^148^C-F2 clone. Immunostaining of ΔN^148^C-F2 and ΔN^148^C-F2; *CDH1 OE* cells were performed before and after Dox treatment for 6 h (upper panels). Antibodies used were specific to E-cadherin (yellow). qRT-PCR and western blot analysis showed *CDH1* mRNA (lower left) and E-cadherin protein levels (lower right), respectively. **G** Nuclear translation (fold) of ΔN^148^C in *CDH1 OE* cells. Immunostaining of ΔN^148^C-F2 clone and ΔN^148^C-F2;C*DH1 OE* cells were performed after Dox treatment for 24 h (upper panels) and at DE (d1) (lower panels), using antibody specific to Flag-tag (red). Areas surrounded by the dashed white lines were enlarged below. **H **Relative expression (fold) of *FOXA2* and *SOX17* at DE (d4) in ΔN^148^C-F2 and the ΔN^148^C-F2;C*DH1 OE* cells. For statistical analysis, ΔN^148^C-F2;C*DH1 OE* clones’ data were compared to that of ΔN^148^C-F2 cells. Nuclei were counterstained using Hoechst (blue) in **A**, **B**, **C**, **D**, **F** and **G**. Scale bars = 50 μm in all images. The qRT-PCR data in **A**, **B**, **C**, **E**, **F**, and **H** were presented as mean ± SD (n = 3). Student’s t-test were performed for statistical analysis. ns, not significant; *, *p* ≤ 0.05; **, *p* ≤ 0.01; ***, *p* ≤ 0.001

We next examined nuclear translocation of the various β-catenin mutants. Indeed, S33Y and ΔN^90^ displayed significantly stronger nuclear accumulation compared to FL β-catenin, either upon Dox treatment or after DE induction (Fig. S5). Interestingly, ΔN^148^ and ΔN^148^C mutants also exhibited significant nuclear translocation (Fig. 3A), though the signals were weaker than S33Y and ΔN^90^. Rather distinctly, ΔC mutant was predominantly detected at cell junctions and remained largely unchanged even after DE induction (Fig. 3A). The analysis of two individual clones carrying ΔN^148^, ΔN^148^C (flag-tagged), and ΔC (flag-tagged) mutants at different expression levels further support a dose-dependent effect. The nuclear translocation of ΔN^148^ and ΔN^148^C were largely correlated with their transcription levels; meanwhile the enhanced localization of ΔC (flag-tagged) at cell junctions was associated with higher expression (Fig. 3A, middle panels). Notably, while the nuclear translocation intensities of S33Y, ΔN^90^, FL, ΔN^148^ and ΔC positively correlated with their TOPFLash activities (Fig. 2B), ΔN^148^C was an exception. The ΔN^148^C mutant showed prominent nuclear translocation upon DE induction, resembling ΔN^148^ (Fig. 3A), but induced low transactivation of TOPFLash reporter like ΔC (Fig. 2B), suggesting a nuclear function distinct from the conventional transactivation dependent on the CTD-TCFs interactions.

To further examine whether the nuclear translocation of ΔN^148^C correlated with its functional role in rescuing DE differentiation, we modulated the transgene expression with different Dox concentrations. We observed a dose-dependent nuclear translocation of ΔN^148^C (Fig. 3B, left panel). Markedly, the levels of nuclear ΔN^148^C signals were positively correlated with the DE rescue outcomes, as indicated by the concurrent activation of *FOXA2* and *SOX17*, as well as other DE-related genes *GATA4*, *GATA6* and *EOMES* at DE (d4) (Fig. 3B, right panel). These results further supported that ΔN^148^C rescued DE through its nuclear activity, which was functioning independently of CTD and traditional β-catenin/TCF-based transactivation.

Furthermore, we examined the effect of manipulating E-cadherin, which could sequestrate β-catenin at cell junctions to influence its intracellular accumulation and nuclear translocation. We CRISPR-engineered the ΔN^148^C-F2 clone to disrupt *CDH1,* the gene encoding E-cadherin (Fig. 3C and Fig. S6). Two *CDH1-/-* clones were obtained and both maintained normal hESC features in the absence of Dox (Fig. 3C). Upon Dox treatment and DE induction, these ΔN^148^C-F2;*CDH1-/-* cells showed significantly enhanced nuclear translocation of ΔN^148^C compared to the parental ΔN^148^C-F2 clone (Fig. 3D). DE induction from the two ΔN^148^C-F2;*CDH1-/-* clones consistently yielded *FOXA2* and *SOX17* expression at higher levels compared to ΔN^148^C-F2 clone, indicating enhanced DE differentiation (Fig. 3E). Oppositely, when Dox-induced *CDH1* overexpression was introduced in the ΔN^148^C-F2 clone via lentivirus, excessive E-cadherin dramatically distorted cell–cell junctions as early as 6 h after Dox treatment (Fig. 3F). Meanwhile, the elevated E-cadherin strongly sequestrated ΔN^148^C to membrane junctions and in cytoplasm, which largely abolished its nuclear translocation (Fig. 3G and Fig. S7) and significantly impeded the DE rescue (Fig. 3H). Altogether, the robust positive correlations among ΔN^148^C expression, nuclear translocation, and DE rescue outcome, supported that the nuclear activity of ΔN^148^C β-catenin was crucial for rescuing DE differentiation.

### Truncated β-catenin ΔN^148^C rescued DE commitment through transactivation of primitive streak genes

To explore the CTD-independent unconventional nuclear function of β-catenin underlying DE differentiation, we performed RNA-seq analysis of the various rescue clones. Transcriptome data were collected at two time points, after Dox treatment (d0) and at DE (d1) (Fig. 1F and Fig. S8A), to probe distinct transcriptional responses. Principal component analysis (PCA) of the profiles at DE (d1) revealed a close similarity among FL, ΔN^148^, ΔN^148^C and ΔC clones, which were collectively disparate from S33Y and ΔN^90^ clones (Fig. S8B). This clustering result highly correlated with the differentiation phenotypes, dividing the rescue clones into DE-potent (DE^high^) and DE-defective (DE^low^) groups. Interestingly, the DE^high^ clones, not DE^low^ clones, were grouped closer with the *CTNNB1-/-* KO#7, suggesting that DE rescue required only low β-catenin activity which had functions distinctive from the conventional Wnt/β-catenin/TCF signaling.

**Fig. 4:**
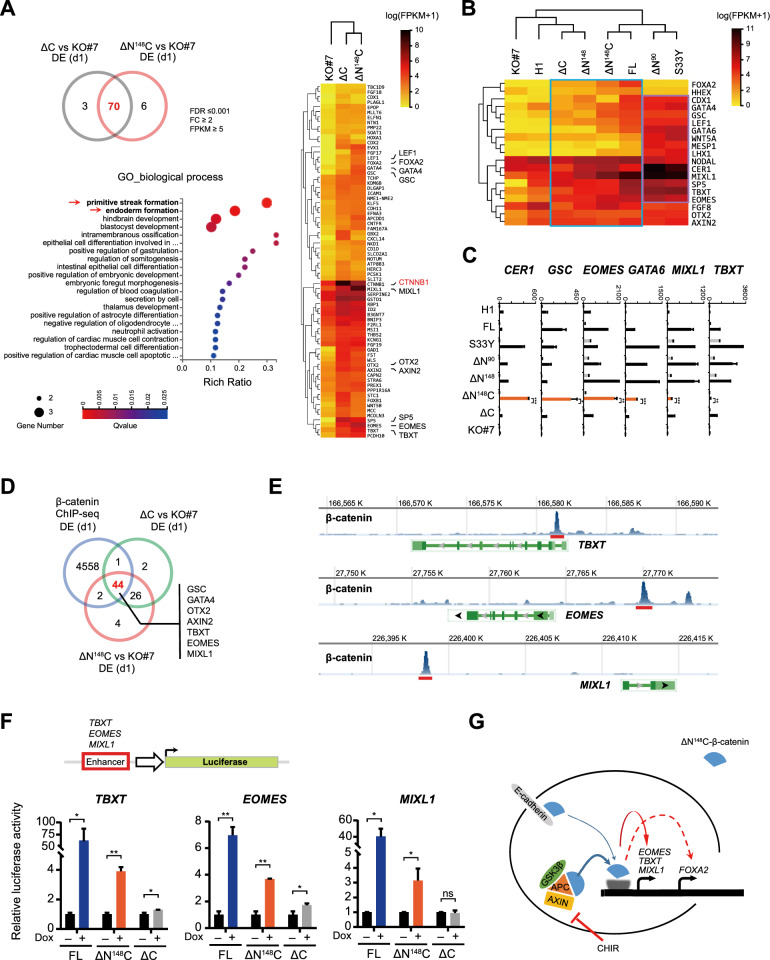
Transactivation of primitive streak genes in ΔN^148^C and ΔC clones upon DE induction. **A** Venn diagram indicated that 70 genes were upregulated in both ΔC and ΔN^148^C clones at DE (d1) compared to *CTNNB1-/-* clone (KO#7) (FDR ≤ 0.001, FC ≥ 2) (upper left). GO enrichment (lower left) and heat map analysis (right) further highlighted transactivation of genes related to primitive streak (PS) and endoderm formation. **B** Heat map showing the normalized expression of 19 PS- and mesendoderm-related in all cell clones examined at DE (d1). The blue box indicates moderate activation of PS genes in FL, ΔN^148^, ΔN^148^C and ΔC clones, while the purple box highlights hyperactivation of PS genes in S33Y and ΔN^90^ clone. **C** Selective qRT-PCR showing relative expression (fold) of PS genes (*TBXT, MIXL1, GATA6, EOMES, GSC,* and *CER1*) at undifferentiated stage (untreated), after Dox treatment (Dox), and at DE (d1). The data values were normalized to undifferentiated wt hESC (H1) and presented as mean ± SD (n = 3). **D** Venn diagram showing genes commonly upregulated in ΔN^148^C and ΔC clones at DE (d1) compared to KO#7 (FDR ≤ 0.001, FC ≥ 2, FPKM ≥ 5) were overlapped with candidate β-catenin target genes previously identified by ChIP-seq in hESCs (GSE182842) [31]. Key PS and endoderm genes highlighted by the overlap were indicated. **E** The binding profiles of β-catenin in *TBXT*, *EOMES*, and *MIXL1* loci in wt hESC (GSE64758) [30]. Red bars indicated the enhancer regions used in reporter analysis. **F** Luciferase reporter analysis of *TBXT*, *EOMES*, and *MIXL1* enhancer activities. Shown were relative luciferase activities detected after transducing the three reporters into FL, ΔN^148^C, ΔC and KO#7 clones, followed by Dox induction. The data values were normalized to KO#7 and presented as mean ± SD (n = 3). For statistical analysis, student’s t-test were performed and all data were compared to non-induced cells. ns, not significant; *, *p* ≤ 0.05; **, *p* ≤ 0.01; ***, *p* ≤ 0.001. **G** Schematic model for β-catenin-mediated transactivation of PS genes during DE commitment. *FDR* false discovery rate, *FC* fold change, *FPKM* fragments per kilobase of transcript per million mapped reads, *GO* gene ontology

We then focused on ΔN^148^C and ΔC clones, both of which lacked the CTD and traditional TCF-mediated strong transactivation activity (Fig. 2A, B). Notably, gene ontology (GO) analysis of the 70 genes commonly upregulated in ΔN^148^C and ΔC clones highlighted the induction of primitive streak (PS) and endoderm (Fig. 4A, left panel, red arrows). Expression heat map further confirmed significant upregulation of multiple mesendoderm genes, including *GSC, GATA4, AXIN2, and EOMES* (Fig. 4A, right). Interestingly, heat map of all examined clones showed that expression levels of the PS and mesendoderm genes [27–29] in FL, ΔN^148^, ΔN^148^C and ΔC clones were positively correlated with the DE rescue outcomes (Fig. 4B, in blue box). Whereas surprisingly, significant or even higher activation of these genes were observed in S33Y and ΔN^90^ clones (Fig. 4B, in purple box), which were contradictory to the poor DE rescue outcomes.

To further clarify the gene activations specifically responsible for CTD-depleted weak β-catenin mutants, we performed real-time RT-PCR. *TBXT, MIXL1,* and *GATA6* exhibited high expressions in FL, S33Y, ΔN^90^ and ΔN^148^ clones but moderate activation in ΔN^148^C and ΔC, indicating transactivation in both CTD-dependent and CTD-independent manners (Fig. 4C). Whereas markedly, *EOMES, GSC, and CER1* showed stronger activation in ΔN^148^C clone than in S33Y and ΔN^90^ clones, indicating evident CTD-independent transactivation (Fig. 4C). These results collectively supported that β-catenin regulated conventional PS/mesendoderm genes via distinct mechanisms.

Furthermore, we compared the genes upregulated in ΔN^148^C and ΔC clones with candidate β-catenin target genes identified by ChIP-seq analysis in hESCs (GSEA58476, GSE64758, GSE182842) [29–31]. Notably, PS and mesendoderm genes, including *TBXT, MIXL1, EOMES,* and *GSC,* were largely highlighted in the overlap gene set (Fig. 4D, E). This coincidence suggested that CTD-independent transactivation could be an intrinsic function of wt β-catenin, which played a pivotal role in activating PS/mesendoderm genes during hESC commitment.

We further constructed luciferase reporters carrying β-catenin-binding enhancers in *TBXT, EOMES,* and *MIXL1* locus [29] and examined whether their activities can be regulated in FL, ΔN^148^C, ΔC, and KO#7 clones. Indeed, all three enhancers of *TBXT, EOMES,* and *MIXL1* were significantly activated by the Dox-induced ΔN^148^C, yielding enhanced luciferase signals (Fig. 4F). Consistent with the expression data (Fig. 4B), FL β-catenin triggered much higher activation of these enhancers compared to ΔN^148^C, whereas much lower reporter activities were detected in ΔC clones (Fig. 4F), correlating with its weaker and delayed DE rescue. These results further supported that β-catenin indeed mediated weak transactivation independent of CTD and such activity was involved in PS gene activation and DE commitment (Fig. 4G).

### The level of β-catenin activity determined the bifurcation of endo- and mesoderm

**Fig. 5:**
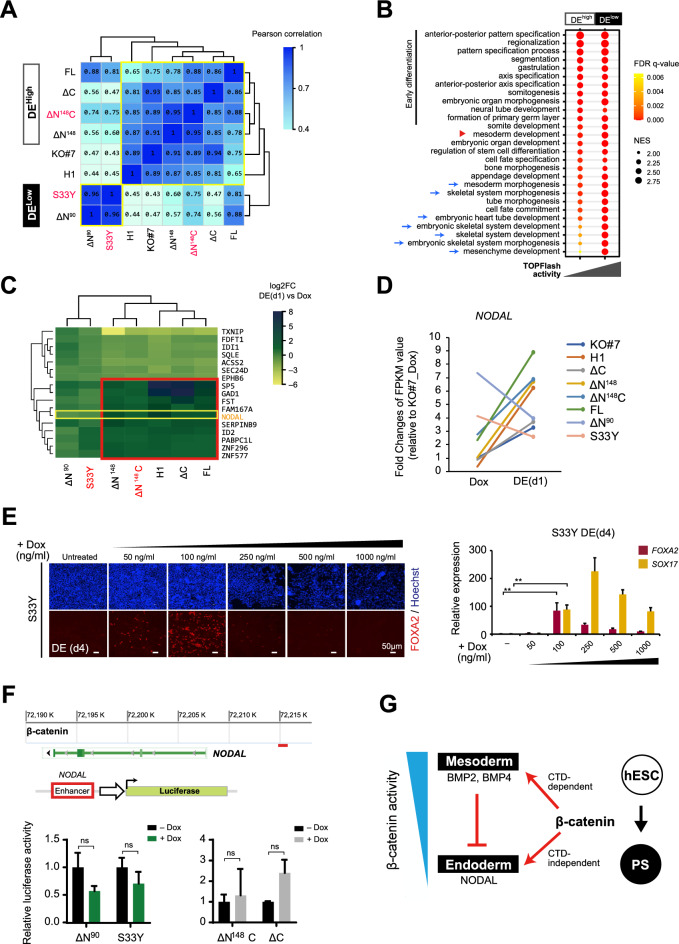
Transactivation of* NODAL* by ΔN^148^C and ΔC β-catenin mutants determined the DE rescue phenotype. **A** Transcriptomic correlation heat map of eight cell clones, based on RNA-seq data at DE (d1) and built with R values. Data are Pearson correlation coefficients. Two clusters were indicated as DE^high^ and DE^low^ based on the DE rescue outcomes. **B** GSEA analysis to determine the difference between DE^high^ and DE^low^ groups. Transcriptomic changes of each DE^high^ and DE^low^ clone were calculated at DE (d1) and subjected to GSEA analysis using GSEA v3.0 and MSigDB C5 database v6.2. Normalized enrichment scores (NES) of the top 20 GO BPs enriched in either DE^high^ or DE^low^ groups were collectively represented in the dot plot. The size of dots indicates NES, while the color corresponds to the FDR-q value. **C** Heat map showing the expression of 17 genes that were consistently up/down regulated among the DE^high^ clones while down/up regulated in the DE^low^ clones at DE (d1) (FDR ≤ 0.001, fold change ≥ 2). The red box indicated the genes upregulated in DE^high^ group. The yellow box indicated *NODAL* expression. **D** The expression changes of *NODAL* in all cell clones upon Dox treatment (Dox) and at DE (d1). The data shown were fold changes of FPKM values compared to KO#7 Dox. **E** DE differentiation of S33Y clone induced with reduced Dox. Left panels showed the immunostaining of FOXA2 (red) at day 4 post DE induction with Dox at different concentrations (indicated). Nuclei were counterstained using Hoechst (blue). Right panel showed the qRT-PCR analysis of *FOXA2* and *SOX17* at DE (d4). The data values were normalized to untreated control and presented as mean ± SD. Student’s t-test were performed. **, *p* ≤ 0.01. **F** Luciferase reporter analysis of *NODAL* enhancer activities. Upper panel presents the β-catenin binding profile in *NODAL* locus in wt hESC (GSE64758) [30]*.* The red bar indicated the enhancer region tested in reporter analysis. Lower panel shows the relative luciferase activities (fold) analyzed after transfecting the reporter into S33Y, ΔN^90^, ΔN^148^C, and ΔC clones, followed by Dox induction. The data values were normalized to KO#7 and presented as mean ± SD (n = 3). Student’s t-test were performed, and all data were compared to non-induced cells. ns, not significant; *, *p* ≤ 0.05; **, *p* ≤ 0.01; ***, *p* ≤ 0.001. G Schematic presentation for the regulatory role of β-catenin activity level in controlling the cell fate bifurcation into mesoderm and endoderm from hESCs. *DE* definitive endoderm, *FDR* false discovery rate

To investigate the mechanisms underlying the distinct DE rescue outcomes in the DE^high^ and DE^low^ groups (Fig. 5A and Fig. S8B), we selected ΔN^148^C and S33Y to represent each group and compared their transcriptome profiles (Fig. S9A). Consistent with their TOPFlash activities (Fig. 2B), GO enrichment of biological processes (BP) related to conventional Wnt/β-catenin signaling was prominently observed in S33Y clone (Fig. S9B, red asterisks in upper panel), but not in ΔN^148^C upon DE induction. Interestingly, GO BPs enriched in S33Y clone were also extensively associated with the development of mesoderm tissues and organs (Fig. S9B, blue arrows in upper panel). Corroborating this observation, marked activation of BMP signaling (Fig. S9B, red arrowheads) and upregulation of BMP target genes *TBXT, CDX2, MESP2, BMP2* and *BMP4* (Fig. S9C, upper panel) were also confirmed, supporting the mesoderm specification [32, 33]. Whereas distinctly, GO BPs enriched in ΔN^148^C clone highlighted PS formation and endoderm development (Fig. S9B, green arrowheads and green arrows in lower panel), while the higher expression of *NODAL, FOXA2* and *GDF3* in ΔN^148^C than S33Y clone (Fig. S9C, lower panel) indicated activation of *Nodal* signaling alongside effective DE rescue [34].

To further explore the functional role of β-catenin underlying the cell fate bifurcation from mesendoderm, we performed gene set enrichment analysis (GSEA) to determine the feature BPs in the DE^high^ and DE^low^ groups (Fig. 5B). Indeed, extensive enrichment of GO BPs related to mesoderm tissue/organ formations was observed in DE^low^ but not DE^high^ group (Fig. 5B, blue arrows). Despite the BP of mesoderm development was significantly enriched in both groups, based on the transcriptomic changes from Dox (d0) to DE (d1) in individual clones (Fig. 5B, red arrowhead), examination of leading edge gene sets revealed much enhanced activation of relevant genes among DE^low^ clones than in DE^high^ clones (Fig. S10A, B, right panels) and distinctive activation of *BMP4, WNT5A, TWSG1, TBX20, OSR1* (Fig. S10C) in the DE^low^ group, which were BMP targets and posterior PS genes associated with pro-mesoderm activities [35–38]. These observations collectively indicated that the hyperactive β-catenin mutant S33Y and ΔN^90^ indeed promoted mesoderm specification in the DE^low^ clones.

Since the GSEA for DE^high^ group uncovered less distinctive GO BP enrichments (Fig. 5B), we examined genes commonly upregulated among DE^high^ clones from Dox (d0) to DE (d1). Despite the varied phenotypes and transcriptomic changes among FL, ΔN^148^C, ΔN^148^, and ΔC clones, 17 genes showed common expression changes (fold change ≥ 2) upon DE induction and 10 were upregulated (Fig. 5C, red box). Markedly, the endoderm driver gene *NODAL* was consistently activated among the DE^high^ clones (Fig. 5C, yellow box), suggesting that the DE rescue phenotypes were induced through the conserved DE differentiation mechanism mediated by Activin/Nodal signaling [33].

Oppositely, in the DE^low^ clones (S33Y and ΔN^90^), evident repression of *NODAL* was observed (Fig. 5D), suggesting that the high β-catenin activity rendered by S33Y and ΔN^90^ might suppress endoderm specification through repressing *NODAL* signaling. To further verify the suppressive role of hyperactivity of β-catenin, we re-examined DE differentiation in the S33Y clone using reduced Dox (Fig. 5E). Indeed, we observed significantly improved DE rescue when Dox concentration was reduced to 100 ng/ml, corresponding to 10% of the original dose. We then constructed the luciferase reporter carrying *NODAL* enhancer and examined in different rescue clones (Fig. 5F, upper and middle panels). Although a trend of repression was observed upon Dox-induction of S33Y or ΔN^90^, the reduction of *NODAL* enhancer activities was not significant (Fig. 5F, lower panels). These results collectively supported that it was the hyperactivity of β-catenin that suppressed endoderm specification in S33Y and ΔN^90^clones, likely through an indirect repression of *NODAL* (Fig. 5G).

## Discussion

In this study, we conducted a systematic analysis of β-catenin’s role in DE differentiation from hESCs. Based on a loss-of-function model generated through CRISPR-based insertional gene disruption, we performed DE rescue to investigate various β-catenin functional mutants. Among them, the truncated β-catenin mutants ΔN^148^C and ΔC effectively rescued the DE-differentiation defect in *CTNNB1-/-* hESCs, despite their deficiency in conventional CTD/TCF-dependent transactivation. Conversely, the hyperactive β-catenin mutants S33Y and ΔN^90^ showed the least rescue potential. Our analysis ruled out enhanced EMT and membrane function of β-catenin as mediators of the DE rescue effect. Instead, we found that the ΔN^148^C mutant possessed CTD-independent transactivation activity, inducing PS/mesendoderm genes during DE induction. Further analysis revealed that S33Y and ΔN^90^ promoted mesoderm formation while suppressing DE formation. Collectively, these findings emphasize the pivotal role of β-catenin in the bifurcation of endo- and mesoderm. Weak to moderate β-catenin activities, represented by ΔN^148^C, ΔC, and ΔN^148^, mediates PS formation and DE commitment, while high β-catenin activity induced by S33Y and ΔN^90^ drives mesoderm formation and represses the endoderm lineage. Our study provides valuable insights into the central role of β-catenin activity levels in early lineage specifications.

## Evolutionarily conserved role of Wnt/β-catenin activation in mesendoderm commitment

Wnt/β-catenin activity is essential to early pluripotent cell differentiation. Studies in mice and frogs with β-catenin deficiency showed that they failed to form meso- and endoderm germ layers and died before gastrulation [39–41]. Similarly, genetic ablations of β-catenin in mESCs resulted in impaired meso- and endodermal differentiation in vitro [13–16]. Recent studies further revealed that depletion of β-catenin in hESCs, either through genetic knockout or shRNA-mediated knockdown, hindered their differentiation into meso- and endodermal lineages [17, 18]. Consistent with these findings, a higher Wnt/β-catenin activity correlated with increased expression of mesendoderm markers in hESC single cells [42]. Activation of Wnt/β-catenin signaling using Wnt ligands or agonists induced PS genes [29] and promoted mesendoderm differentiation from hESC [18, 42–44]. Our results from the loss-of-function and rescue analyses in new hESC models are consistent with these previous observations, highlighting the evolutionarily conserved requirement of Wnt/β-catenin activity for mesendoderm commitment.

### The membrane vs nuclear functions of β-catenin during DE induction

Β-catenin is a bi-functional protein that forms complex with cadherins to maintain the integrity of adherens junctions and cell–cell contacts for hESC survival [45]. During hESC differentiation into mesendoderm, disassembly of E-cadherin/β-catenin complex is associated with the EMT process, leading to a characteristic colony dispersal phenotype [24, 46].

Our findings revealed that ΔN^148^C and ΔN^148^ mutants disrupted cell–cell contacts, leading to cell scattering and disassembly of hESC colonies. Depletion of α-catenin binding domain in β-catenin abolished its connection to cytoskeleton (F-actin) and rendered a dominant-negative effect [7, 8]. Hence, ΔN^148^C and ΔN^148^ interfered with the interaction between E-cadherin and JUP and disrupted existing cell–cell contacts in the *CTNNB1-/-* hESCs. Interestingly, we observed that the cell scattering phenotype was not associated with enhanced EMT, which is known to promote DE formation [24] (Fig. 2G). Given the robust DE rescue by ΔN^148^C and ΔN^148^, these results suggested that the membrane function of β-catenin was not required in DE differentiation.

Markedly, we observed dose-dependent nuclear translocation of ΔN^148^C and ΔN^148^, and their nuclear levels were positively correlated with rescue of DE differentiation (Fig. 3). This indicated that the ΔN^148^C preserved a nuclear activity similar to ΔN^148^ in mediating the DE rescue, despite lacking the CTD and traditional TCF-dependent transactivation capability. The detection of DE rescue in ΔC clones further supported this notion, while the hyperactive S33Y and ΔN^90^ mutants exhibited the least DE rescue. These findings suggest that ΔN148C and ΔN148 mutants possess non-conventional β-catenin activity responsible for mediating the DE commitment.

### PS genes are induced by weak β-catenin activity through CTD-independent transactivation

TCF/LEFs are well-known transcription factors that partner with nuclear β-catenin to activate target genes, driving wide-ranging biological processes during embryonic development and tissue homeostasis in response to Wnt signaling. However, in addition to the TCF/LEF-involved predominant transactivation, recent studies have indicated that β-catenin can also mediate weaker transcriptional regulations, which have often been overlooked. Doumpas et al. analyzed HEK293T cells lacking all four TCF/LEF genes and found that certain β-catenin target genes can be activated without TCF/LEF factors [19]. Similarly, Chen et al. showed that low Wnt/β-catenin activity could support the differentiation of mESCs in embryoid bodies, independently of TCF signaling and CTD domain in β-catenin [47]. Additionally, β-catenin in hESC was found to bind lineage-specific enhancers without the co-occupancy of TCFs [31].

In our study, we presented new evidence highlighting the importance of weak and CTD-independent nuclear activity of β-catenin in DE commitment. Our findings showed that the β-catenin mutants lacking CTD domain and TCF-dependent transactivation activity, such as ΔN^148^C and ΔC, induced PS genes and rescued the DE differentiation in *CTNNB1-/-* hESCs. Moreover, consistent with previous reports indicating that transient Wnt stimulation is optimal for DE induction [9, 11, 48], we observed robust DE rescue when FL and mutant β-catenin were transiently induced at the initiation stage, from Dox (d0) to DE (d1) (Figs. 1F and 2C). These observations collectively support the notion that the early-responsive genes crucial for DE induction are activated by weak β-catenin with CTD-independent nuclear activity.

### The fine-tuned β-catenin activity controls the cell fate bifurcation towards meso- and endoderm

Our transcriptomic analysis using distinctive rescue clones provided new insights into the importance of finely regulated Wnt/β-catenin activity in determining the lineage choice between endoderm and mesoderm. The DE formation during vertebrate gastrulation has been robustly marked by evolutionarily conserved activation of *FOXA2* and *SOX17*, which requires both Wnt/β-catenin and Nodal signaling [49, 50]. Previous studies have found that in vitro DE differentiation from hESCs can be induced by transient Wnt stimulation and high levels of Activin A, without activating mesoderm markers [27]. Conversely, high levels of Wnt/β-catenin activity favor the activation of BMP signaling while repressing Nodal signaling, thereby promoting mesoderm formation while inhibiting endoderm [33]. However, the mechanisms underlying the modulation of weak and strong β-catenin activities remain poorly understood, leaving several crucial regulatory processes unexplained.

In our study, we observed that hyperactive β-catenin mutants S33Y and ΔN^90^ impaired DE induction and resulted in poor rescue, despite high activation of many PS genes (Fig. 4B). Further analysis revealed repression of *NODAL* expression, along with high activation of BMP signal and mesoderm genes in the S33Y and ΔN^90^ clones (Fig. 5 and Fig. S10), indicating that high transactivation through the conventional CTD/TCF axis promotes mesoderm formation. In contrast, the β-catenin and mutants with weak-to-moderate activities, namely FL, ΔN^148^C, ΔN^148^, and ΔC, exhibited robust DE rescue phenotypes associated with consistent activation of *NODAL* and low BMP signaling (Fig. 5 and Fig. S10). Among these DE^high^ variants, FL and ΔN^148^ carried intact CTD, while ΔN^148^C and ΔC mutants lacked the CTD. These observations collectively supported the notion that β-catenin possesses an intrinsic property to convey the weak-to-moderate activity required for endoderm formation. Indeed, a recent in vitro modeling of peri-implantation development using single-cell technology has largely corroborated this finding [51]. The non-conventional β-catenin transactivation represents a distinct regulatory mechanism that may be involved in a range of underexplored biological processes.

### Limitations of this study

In this study, we focused on evaluating the rescue phenotypes and functions of β-catenin mutants in the H1 hESC line by examining the expression of classical PS/mesendoderm and DE marker genes. However, it is important to note that our data may not fully recapitulate the comprehensive process of human endodermal development due to the intrinsic limitations of the hESC model and the analysis methods employed. Additionally, our DE differentiation was induced in a 2D-culture system, which may not fully reflect the complexity of in vivo development. Therefore, future investigations should consider validating our findings in advanced models such as human embryoids to further explore and confirm these observations.

Furthermore, β-catenin is known for its dynamic conformation and complex interactions with multiple co-factors. To gain a deeper understanding of β-catenin's functions, it would be valuable to conduct detailed structural comparisons among different β-catenin mutants and perform integrative analysis of the protein interactome network. This effort would help identify crucial functional co-factors and regulators of β-catenin and provide insights into the mechanisms underlying DE commitment.

## Conclusion

In summary, our study on various β-catenin mutants in rescuing DE differentiation from hESCs have revealed an unconventional regulatory function of β-catenin through weak transactivation, emphasizing that the levels of β-catenin activity play a central role in determining the lineage choice between endoderm and mesoderm. These findings contribute to a better understanding of the transcriptional regulatory mechanisms of β-catenin in early lineage specification and have broad implications for manipulating hESC differentiation.

### Supplementary Information

Supplementary Material 1.

## Data Availability

All data generated or analyzed during this study are included in this manuscript and its supplementary information files.
