# Supplementary material for: β-catenin mediates endodermal commitment of human ES cells via distinct transactivation functions
Source: Cell Biosci. 2024 Jul 24;14:96. doi: 10.1186/s13578-024-01279-5 (PMC11267888; doi:10.1186/s13578-024-01279-5)
Supplement: Supplementary file 1 — Supplementary Material 1. [file 13578_2024_1279_MOESM1_ESM.pdf]

Supplemental Information

β-catenin mediates endodermal commitment of human ES cells via distinct transactivation functions

Xun Ma*, Liujiang Dai*, Chunlai Tan*, Jiangchuan Li, Xiangjun He, Yaofeng Wang, Junyi Xue, Min Huang, Jianwei Ren, Yin Xia, Qiang Wu, Hui Zhao, Wai-Yee Chan, Bo Feng

Supplemental Methods

Transfection

Prior to transfection, H1 hESC colonies were digested using TrypLE™ (Gibco) and dissociated into single cells in presence of 10 µM Rock inhibitors, Y-27632 (STEMCELL Technologies). Total 0.8 µg plasmids were used to transfect 2 × 10^5 cells of H1 hESCs using FuGENE® HD Transfection Reagent (Promega) and seeded into one 24-well in mTeSR1 supplemented with 10 µM Y-27632. Transfected hESCs were maintained for 2–3 days with medium refreshed daily, before subsequent analysis by Fluorescence-activated Cell Sorting (FACS) analysis, qRT-PCR, or Luciferase assay.
HEK293T cells were seeded at a density of 2 × 10^5 cells/well in 24-well plates, at 12 hrs before transfection. Total 0.8 µg plasmids were used to transfect the cells in each well, using Lipofectamine 3000 (Life Technologies) following the manufacturer's instruction. Transfected HEK293T cells were maintained for 2 days before FACS analysis, qRT-PCR, or Luciferase assay.

Luciferase assay

For TOPFlash reporter assay in **Fig. 2B**, the plasmid encoding a specific β-catenin mutant (50 ng) was co-transfected together with 7×TCF TOPFlash luciferase reporter (50 ng) and Renilla plasmid (Addgene #118016) (20 ng) into 2 x 10^4 *CTNNB1-/-* HEK293T cells (clone #8) in one well of 96-well plates, using Lipofectamine 3000 (Thermo Fisher Scientific). All analyses were performed in triplicates. The medium was refreshed after 12 hrs.
For enhancer analysis in **Fig. 4F** and **Fig. 5E**, the reporter construct carrying a specific enhancer (100 ng) was co-transfected with the Renilla plasmid (40 ng) into 2 x 10^4 hESCs of a rescue clone, in one well in 48-well plates. The transfection was performed using FuGENE® HD Transfection Reagent (Promega), in mTeSR1 medium supplemented with 10 µM Y-27632 and 0.5 µg/ml Dox. All analyses were performed in triplicates. Medium were refreshed daily.
In both TOPFlash and enhancer assays, cell samples were analyzed at 48 hrs post-transfection to detect luciferase activities using Dual-Luciferase Reporter Assay System (Promega). The signals were measured using SpectraMax i3x microplate reader (Molecular Devices), following the manufacturer's instructions. Relative luciferase activity was determined by normalizing the firefly luciferase activity based on Renilla activity.

qRT-PCR

Total RNA from cell samples was extracted using TRIzol reagent (Lige Technologies). 500-1000 ng RNA was reverse transcribed into cDNA using High-Capacity cDNA Reverse Transcription Kit (Applied Biosystems). Quantitative real-time PCR (qRT-PCR) was performed using Power SYBR Green PCR Master Mix (Applied Biosystems) in QuantStudio™ 7 Flex Real-Time PCR system (Applied Biosystems). The primers used for qRT-PCR in this study were listed in Supplementary Table 1.

Flow cytometry

Fluorescence-activated cell sorting (FACS) analysis and cell sorting were performed using BD FACSAria™ II system (BD Biosciences), based on GFP or TdTomato expression. Undifferentiated hESCs (wt H1, *CTNNB1-/-* cells, or rescue clones) were dissociated into single cells using TrypLE (Gibco). The cells were then resuspended in fresh mTeSR1 medium supplemented with 10 µM Y-

27632 and used directly for FACS analysis or cell soring.

Immunofluorescence
Immunofluorescence staining was performed as previously described (1). Basically, hESCs (wt H1, *CTNNB1-/-* cells, or rescue clones) or differentiated cell samples were fixed in 4% PFA/PBS for 10 min and permeabilized in 1% Triton X-100/PBS for 20 min at room temperature. Non-specific binding was blocked using 10% normal goat serum (Sigma) in 0.1% Tween-20/PBS. The samples were then incubated with primary antibody diluted in blocking solution at 4°C overnight, followed by incubation with secondary antibodies at room temperature for 2 – 4 hours. The antibodies used were anti-β-catenin (Santa Cruz, sc-7963), anti-Flag-tag (sigma, F1804), anti-FOXA2 (Santa Cruz, sc-101060), anti-SOX17 (R&D systems, AF1924) anti-OCT4 (Santa Cruz, sc-5279), anti-SOX2 (Santa Cruz, sc-17320), anti-NANOG (R&D systems, AF1997-SP), anti-α-catenin (ENZO, ALX-804-101-C100), anti-E-Cadherin (Cell Signaling Technology, 3195S), anti-JUP (BD Biosciences, 610254), and Alexa Fluor 555- or 647-conjugated secondary antibodies (Thermo Fisher Scientific). F-actin was stained using Alexa Fluor 647-conjungated Phalloidin (Thermo Fisher Scientific). Nuclei were counterstained with Hoechst (Thermo Fisher Scientific).

Western blot
Cells were collected and washed with cold PBS, followed by lysis in cold lysis buffer containing 20mM Tris, 137mM NaCl, 1% Triton X-100, 5 mM EDTA, and Protease Inhibitor Cocktail (Roche) on ice for 20 min. After centrifugation at 4°C for 15 min, protein concentration in supernatants were determined using Pierce™ BCA Protein Assay Reagent (Thermo Scientific™).10 μg protein from each sample was resolved by SDS/PAGE and subsequently transferred to polyvinylidene difluoride (PVDF) membranes (Bio-Rad). The PVDF membranes were blocked with 5% non-fat dry milk in TBST buffer for 1 hr at room temperature and then incubated with primary antibodies for overnight. In next day, the membranes were washed three times with TBST buffer and incubated with HRP-conjugated secondary antibodies at room temperature for 2 – 4 hours. Luminescence signals were detected using Amersham ECL select western blotting detection kit (GE Health Care Life Sciences) and exposed to Super RX-N film (Fuji).
The antibodies used were anti-E-Cadherin (Cell Signaling Technology, 3195S), anti-β-catenin (Santa Cruz, sc-7963), anti-α-catenin (ENZO, ALX-804-101-C100), anti-JUP (BD Biosciences, 610254), anti-Flag (sigma, F1804), anti-β-actin (Santa Cruz, sc-47778), and HRP-conjugated anti-mouse (Cell Signaling Technology, 7076) and HRP-conjugated anti-rabbit (Cell Signaling Technology, 7074).

RNA-seq & Bioinformatics analysis
Total RNA was extracted from wt hESC (H1), *CTNNB1-/-* KO#7 and various rescue clones after Dox treatment for 24 hrs or DE induction for one day (Supplementary Table 2) using TRIzol reagent (Thermo Fisher Scientific) and sent to Beijing Genomics Institute (BGI) for RNA sequencing. Fragment Analyzer and Standard Sensitivity RNA Analysis Kit (15 nt) (DNF-471) were used to verify the RNA quality (RIN/RQN > 8.0, 28S/18S > 2.2) before the construction of cDNA library. RNA sequencing was then performed using DNBSEQ platform (https://www.bgi.com/us/dnbseq-ngs-technology/). SOAPnuke software developed by BGI (Version v1.5.2, https://github.com/BGI-flexlab/SOAPnuke) (2, 3) was used for data filtering to remove the reads containing adaptors or with “N” greater than 5%. Hierarchical Indexing for Spliced Alignment of Transcripts (HISTAT) is used for mapping RNA-seq reads to human genome GRCh38.p12 (4). Bowtie2 (5) was used to map the clean reads to the reference gene sequence (transcriptome), and RSEM (6) was used to calculate the gene expression levels in each sample.
The Dr. Tom online platform (https://www.bgi.com/global/dr-tom/) developed by BGI and R package was used for bioinformatics analysis and visualization. The normalized data of filtered genes in all 18 samples, presented with FPKM (Fragments Per Kilobase Million), were used for principal component analysis (PCA) with the ‘ggplot2’ package in R. To control the data quality, the genes showing FPKM value lower than 5 in all samples were excluded before further analysis. P-value was corrected by multiple hypothesis test and determined the domain value by controlling the FDR (False Discovery Rate). Differentially expressed genes (DEGs), with an FDR $\leq$ 0.001 and fold change $\geq$ 2, were determined using R package DEGseq based on the FPKM values. The R package heatmap was used for hierarchical clustering analysis on the union set differential genes. The R package corrplot was used for sample correlation analysis. Venn diagrams were generated via VENN/UpSetR. The DEGs from selected samples were further examined by gene ontology (GO) enrichment and KEGG pathway enrichment.
The Gene Set Enrichment Analysis (GSEA) was performed using Molecular Signatures Database

v6.2 (MSigDB). For Filtering threshold, we set the Max size (the maximum number of genes included in a pathway) as 500 and the Min size (the minimum number of genes included in a pathway) as 15. The differentiation outcomes observed were defined as DE^{high} and DE^{low} to represent the biological phenotypes. The *CTNNB1-/-* KO#7 sample was defined as non-DE control in this analysis. In Fig. 5B and Supplementary Fig. S10, we compared the transcriptomes of individual DE^{high} or DE^{low} clones to KO#7.

Statistical analysis

All statistical analyses were performed using Excel or GraphPad Prism v9 software. All data (luciferase activity, mRNA expression levels, etc.) were reported as mean ± standard deviation (SD). Student's t-test were used to compare data from two groups. $p < 0.05$ was considered to be statistically significant. *, $p \leq 0.05$; **, $p \leq 0.01$; ***, $p \leq 0.001$.

References:

1. He X, Tan C, Wang F, Wang Y, Zhou R, Cui D, et al. Knock-in of large reporter genes in human cells via CRISPR/Cas9-induced homology-dependent and independent DNA repair. Nucleic Acids Res. 2016;44(9):e85.
2. Cock PJ, Fields CJ, Goto N, Heuer ML, Rice PM. The Sanger FASTQ file format for sequences with quality scores, and the Solexa/Illumina FASTQ variants. Nucleic acids research. 2010;38(6):1767-71.
3. Chen YX, Chen YS, Shi CM, Huang ZB, Zhang Y, Li SK, et al. SOAPnuke: a MapReduce acceleration-supported software for integrated quality control and preprocessing of high-throughput sequencing data. Gigascience. 2017;7(1).
4. Kim D, Langmead B, Salzberg SL. HISAT: a fast spliced aligner with low memory requirements. Nature methods. 2015;12(4):357-60.
5. Langmead B, Salzberg SL. Fast gapped-read alignment with Bowtie 2. Nature methods. 2012;9(4):357-9.
6. Li B, Dewey CN. RSEM: accurate transcript quantification from RNA-Seq data with or without a reference genome. BMC bioinformatics. 2011;12(1):1-16.

Supplemental Figures

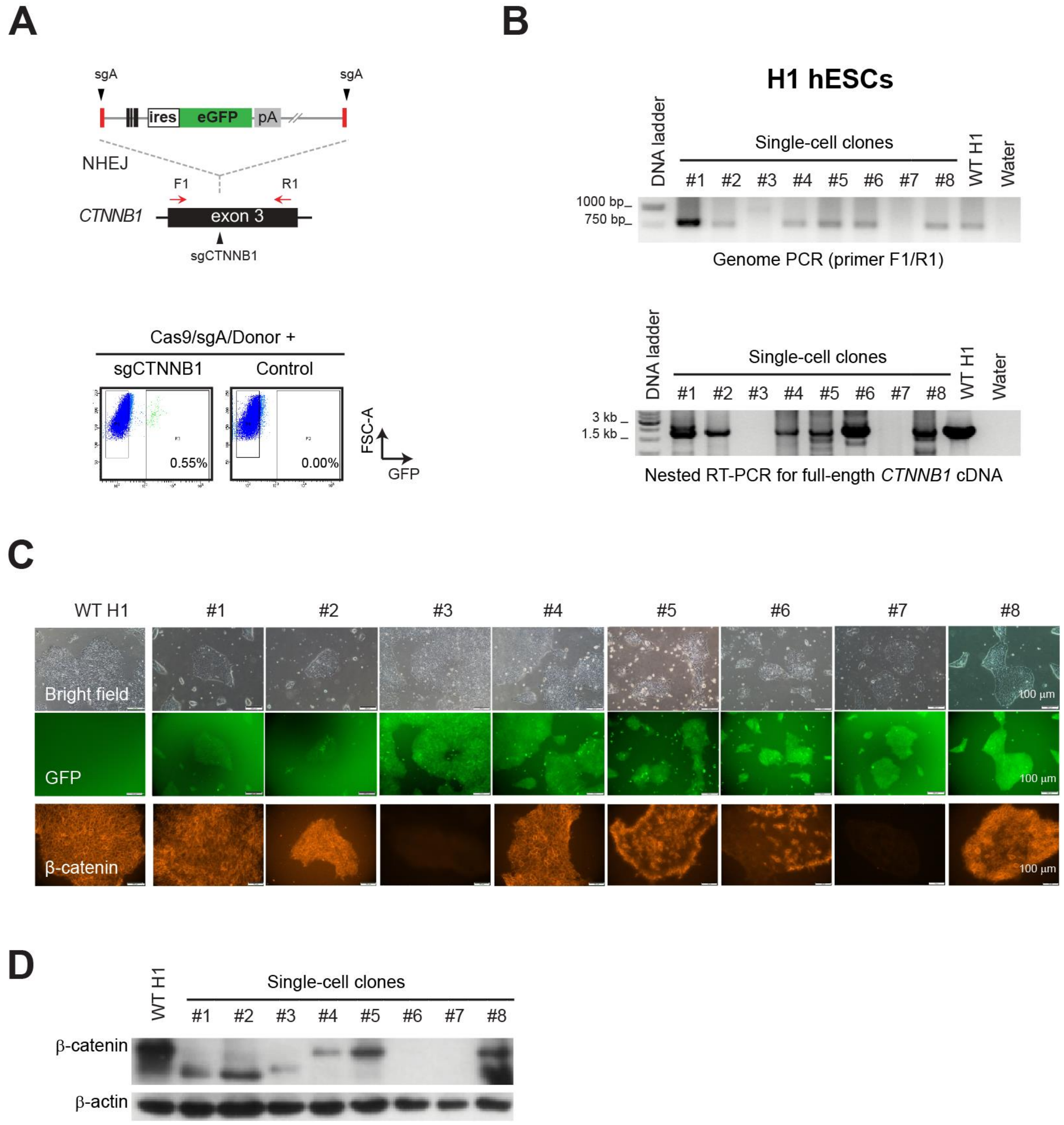

Supplementary Fig. S1. Generation of *CTNNB1-/-* hESCs through CRISPR-mediated insertional gene disruption approach based on NHEJ mechanism (related to Fig. 1).

A. Schematics of the donor plasmid and CRISPR/Cas9-based targeting strategy for NHEJ-mediated insertional disruption of *CTNNB1* gene (upper). FACS analysis of the H1 hESCs edited for *CTNNB1* gene disruption (lower). GFP positive cells and percentage were indicated in each plot inside the right box.

B. Verification of *CTNNB1* gene disruption and β-catenin in single cell clones raised from the edited H1 hESCs, through genome PCR (upper) and nested RT-PCR (lower).

C. Bright field images (upper), GFP signals (middle) and immunostaining of wt hESC (H1) and the *CTNNB1-/-* clones using antibody specific to β-catenin (lower). Scale bars = 100 μm.

D. Western blot analysis of wt hESC (H1) and the *CTNNB1-/-* clones. Antibodies used were specific to β-catenin and β-actin.

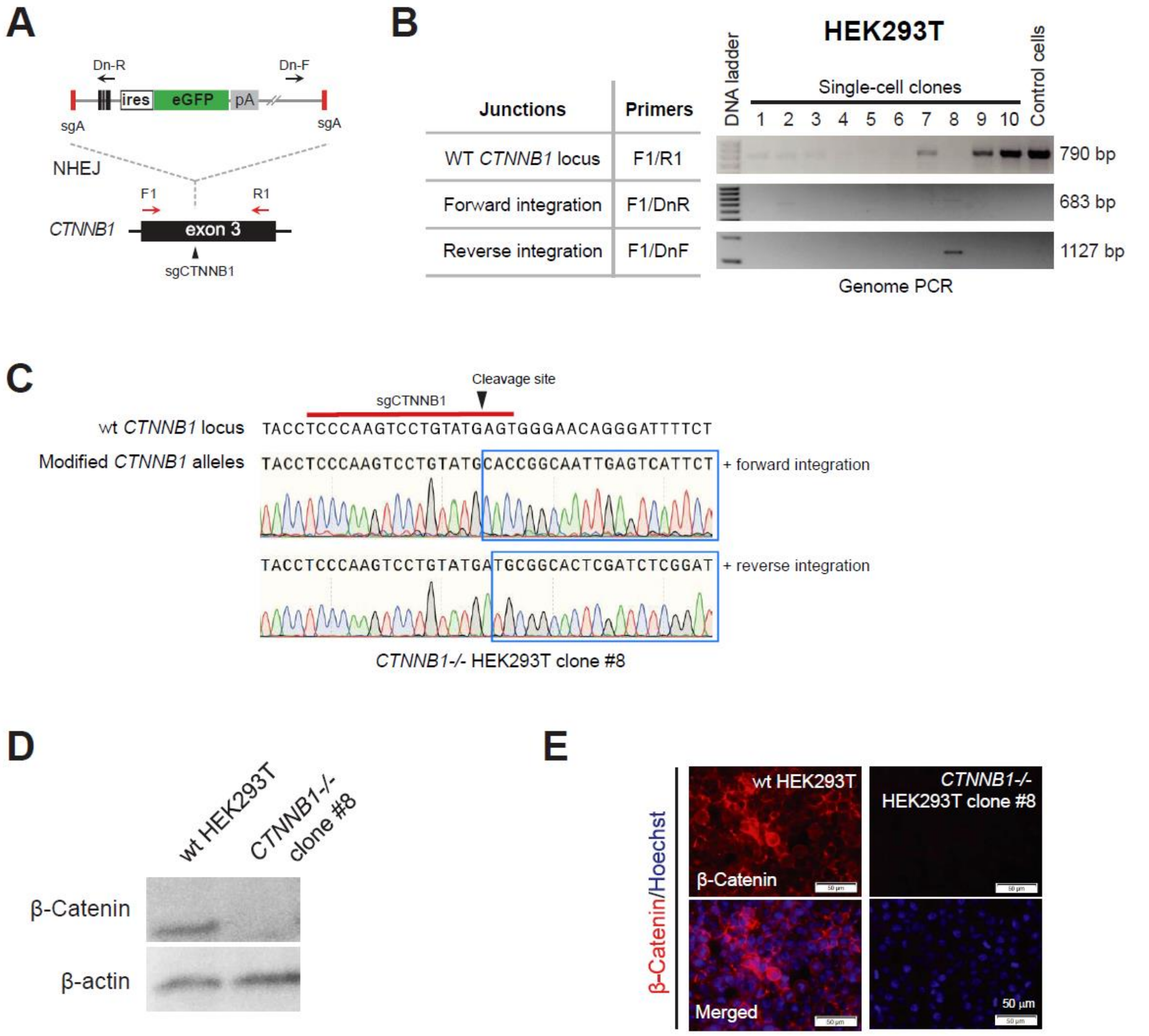

Supplementary Fig. S2. Generation of *CTNNB1-/-* HEK293T cells through the CRISPR-mediated insertional gene disruption approach (related to Fig. 2B)**.**

A. Schematics for CRISPR/Cas9-based insertional disruption of *CTNNB1* gene.

B. Genome PCR of single-cell clones raised from the HEK293T cells transfected for *CTNNB1* gene disruption. The binding sites of three primer pairs used in PCR were indicated in **A**.

C. Sequences of the sgCTNNB1 target region in the modified *CTNNB1* alleles in clone #8. The wt *CTNNB1* locus and sgCTNNB1 target sequences were presented as reference (upper). The graphs showed sequences covering the insertion junctions amplified from the clone #8 in **B**. The blue boxes indicate the donor sequences that have been inserted in two different alleles, with one in the forward (upper) and the other one in the reverse (lower) orientation.

D. Western blot analysis of wt HEK293T and the clone #8 *CTNNB1-/-* HEK293T cells. Antibodies used were specific to β-catenin and β-actin.

E. Immunostaining of wt HEK293T and the clone #8 cells using antibody specific to β-catenin. Nuclei were counterstained using Hoechst. Scale bars = 100 μm.

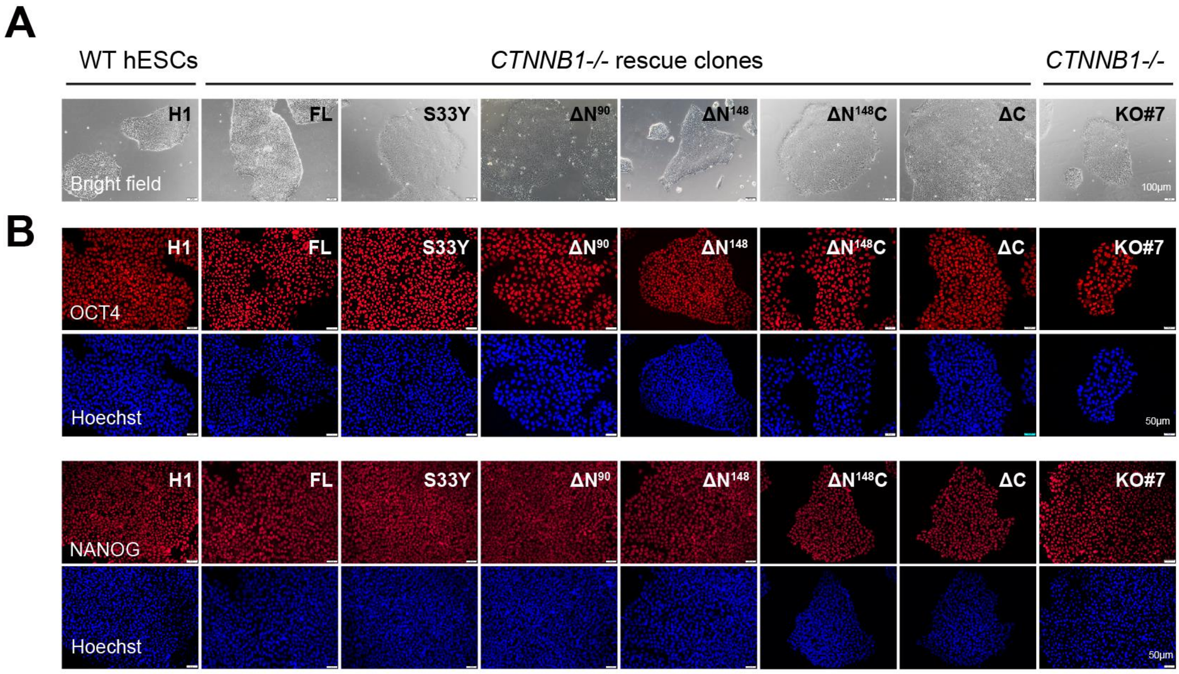

Supplementary Fig. S3. Single-cell clones carrying different β-catenin mutants in the *CTNNB1-/-* hESC (KO#7) background (related to Fig. 2C)**.**

A. Bright-field images showing colony morphologies for the single-cell clones raised from the CTNNB1-/- hESC (KO#7) clone transduced with different β-catenin mutants. Scale bars = 100 μm.

B. Immunostaining of the single-cell clones in A. Antibodies used were specific to pluripotency marker OCT4 (upper panels) and NANOG (lower panels). Nuclei were counterstained using Hoechst. Scale bars = 50 μm.

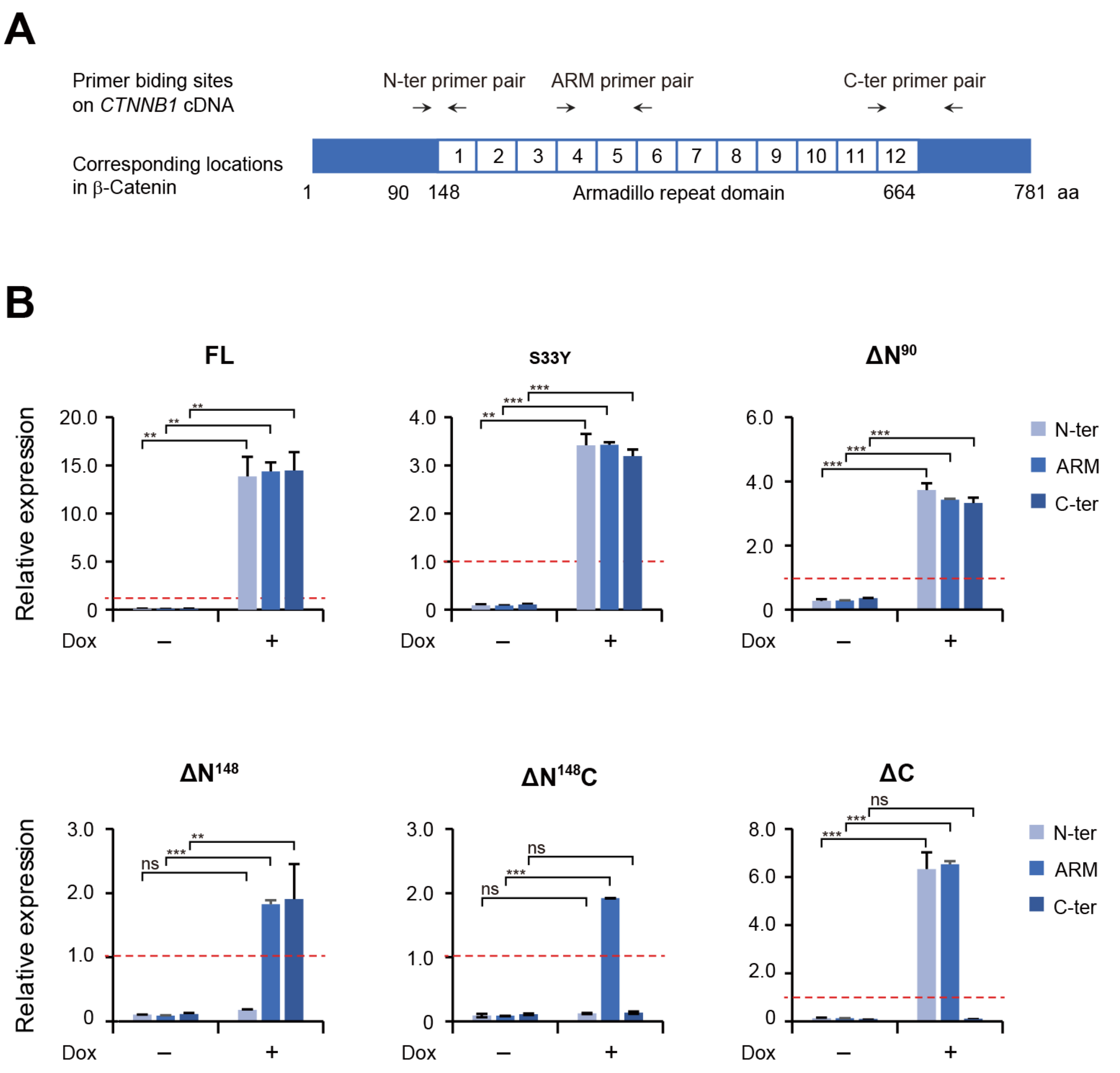

Supplementary Fig. S4. Dox-induced expression of various β-catenin mutant transgenes in single-cell clones (related to Figs. 2C–F)**.**

A. Schematics of β-catenin protein and corresponding locations of the binding sites by three pairs of primers used for qRT-PCR verification of different β-catenin mutants.

B. Analysis by qRT-PCR to verify the dox-induced expression of CTNNB1 mutants in different single-cell clones. Each chart showed the qRT-PCR data for a representative single clone, which was maintained in mTeSR1 medium, with or without Dox induction. The specific CTNNB1 mutant transgene delivered by lentivirus transduction was indicated. The data values were normalized to that in wt H1 hESCs (dashed red lines) and presented as mean ± SD (n=3). The comparison was made for Dox induced sample to non-induced sample. The data values were normalized to wt hESCs (H1) (dashed red lines). ns, not significant; *, $p \le 0.05$; **, $p \le 0.01$; ***, $p \le 0.001$.

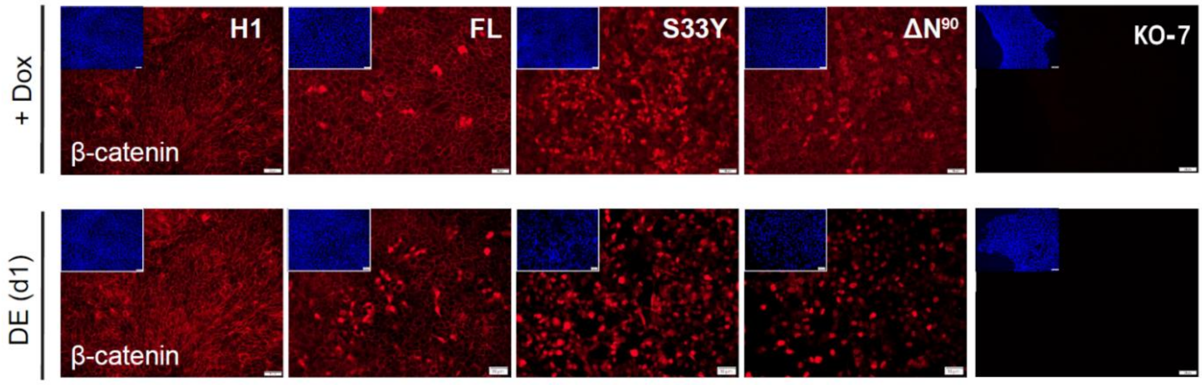

Supplementary Fig. S5. Nuclear translocation of FL β-catenin and S33Y, ΔN^{90} mutants upon Dox treatment and DE induction (related to Fig. 3)**.**
Shown were immunostaining of the wt H1, KO#7, and rescue clones carrying FL β-catenin or S33Y, ΔN^{90} mutants, upon Dox treatment (d0) and at DE (d1). The antibody used was specific to β-catenin. Nuclei were counterstained using Hoechst. Scale bars = 50 µm.

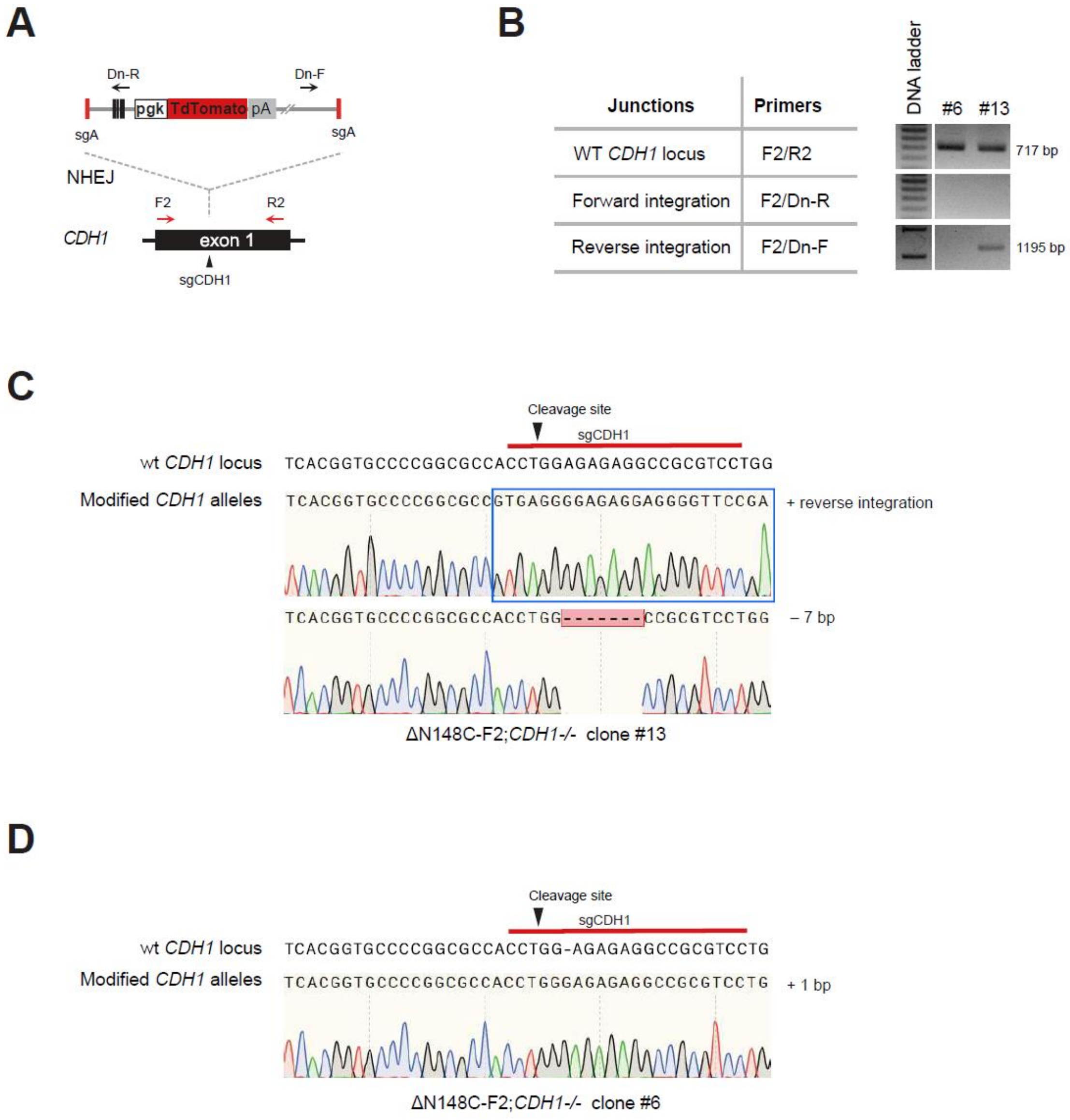

Supplementary Fig. S6. Generation of ΔN^{148}C;*CDH1-/-* hESCs through CRISPR-mediated insertional gene disruption (related to Fig. 3C)**.**

A. Schematics of the donor carrying ires-TdTomato and the targeting strategy for CRISPR-mediated insertional disruption of *CDH1* gene.

B. Genome PCR for single-cell clones raised from the ΔN^{148}C-F2 clone (Fig. 3A), after it was further edited for *CDH1* gene disruption. The binding site of primers used were indicated in **A**.

C. Sequences of the sgCDH1 target region in the modified *CDH1* alleles from the clone #13 in **B**. The wt *CDH1* locus and sgCDH1 target sequence were presented as reference (upper). The graphs showed sequences covering the insertion site or insertion junction amplified from the clone #13 in **B**. The blue boxes indicate the reversely inserted donor sequences in one *CDH1* allele (middle). The other *CDH1* allele was found to carry a deletion of –7 bp at the target site (lower).

D. Sequences of the sgCDH1 target region in the modified *CDH1* alleles in the clone #6 in **B**. The wt *CDH1* locus and sgCDH1 target sequence were presented as reference (upper). The graph showed sequences covering the sgCDH1 targeting site amplified from the clone #6 genome in **B**. Only one type of modification was detected, which contained 1 bp insertion at the target site (lower).

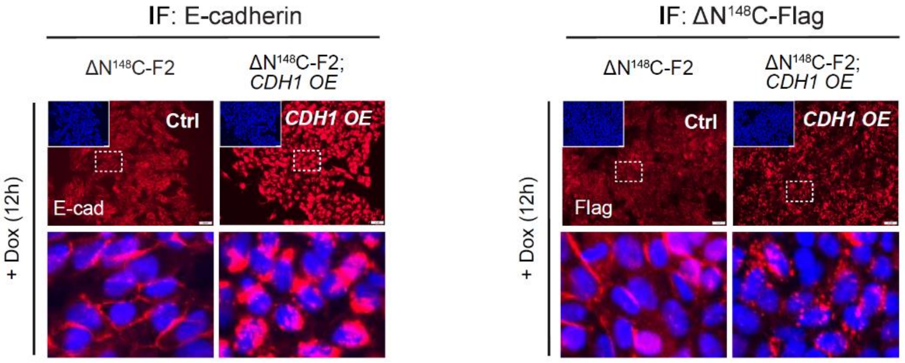

Supplementary Fig. S7. E-cadherin overexpression and its influence on ΔN^{148}C nuclear translocation upon Dox treatment (related to Fig. 3G)**.**
Immunostaining of ΔN^{148}C-F2 and ΔN^{148}C-F2;*CDH1 OE* cells, with Dox treatment for 12 hrs. Shown were images stained using antibodies specific to E-cadherin (left panels, red) and Flag-tag (right panels, red). The areas inside the dashed boxes in upper images were further enlarged and shown below. Nuclei were counterstained using Hoechst (blue). Scale bars = 50 μm.

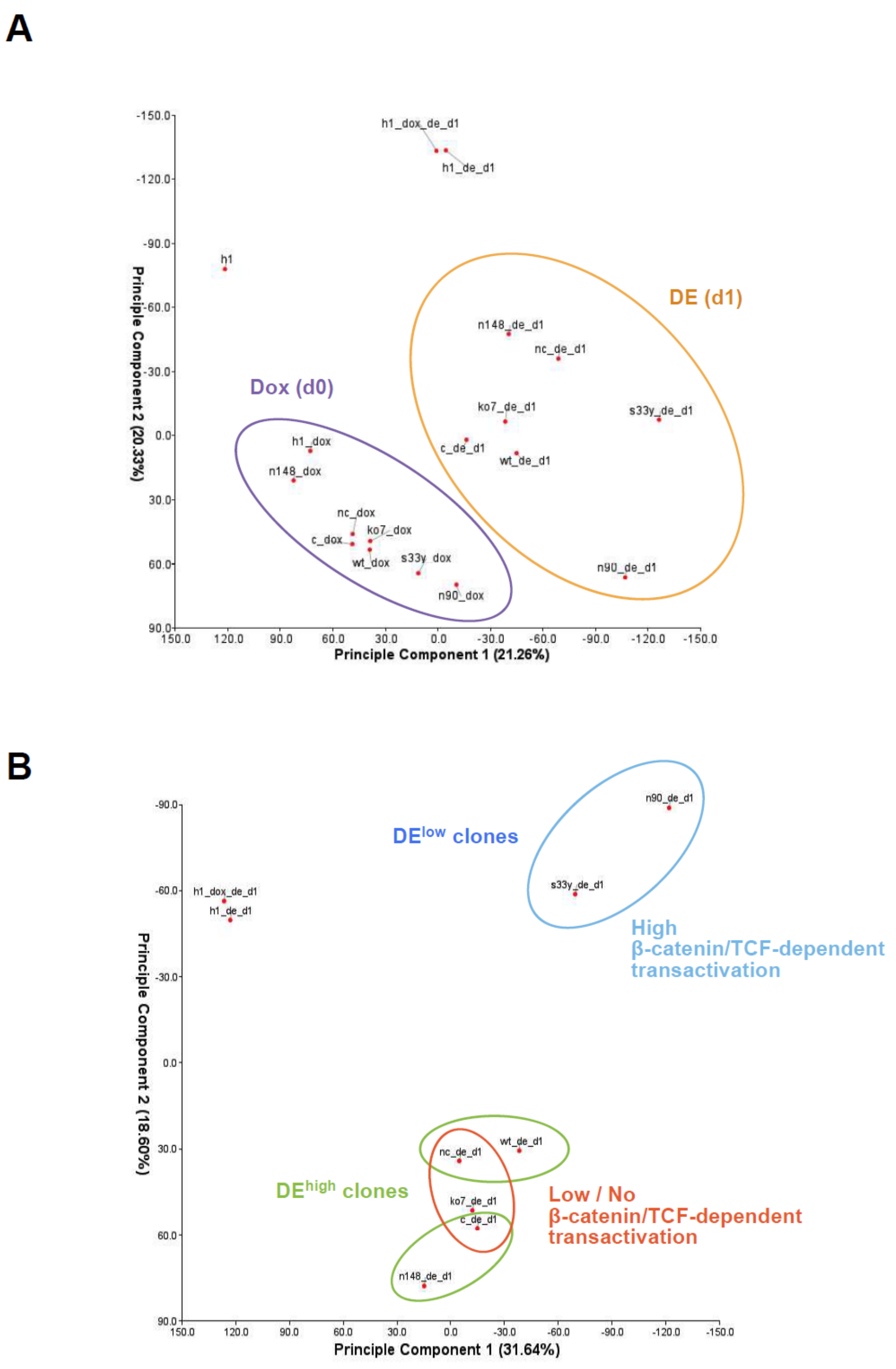

Supplementary Fig. S8. Principal component analysis (PCA) of the RNA-Seq data (related to Fig. 4)**.**

A. PCA plot for the normalized RNA-seq data collected from the all the examined samples, including the various rescue clones, wt H1 hESCs and *CTNNB1-/-* KO#7 cells, treated with Dox (d0) or at DE (d1) (Supplementary Table 2). The samples with Dox treatment (d0) and those collected at DE (d1) formed two large clusters, indicated by the purple circle and the orange circle, respectively. The data for undifferentiated wt hESC (H1) and wt H1 at DE (d1) with or without Dox treatment were located outside of these circles.

B. PCA plot for the RNA-seq data collected from samples at DE (d1). The two light green circles indicated the samples showing significant DE rescue. The light blue circle indicated the samples carrying dominant active β-catenin mutants, displaying high CTD-dependent transactivation activities. The reddish orange circle indicated samples carrying β-catenin knockout or mutants

that showed moderate or low CTD-dependent transactivation activity.

A

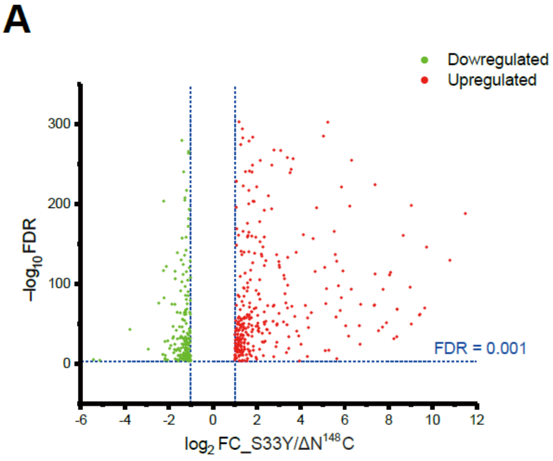

B

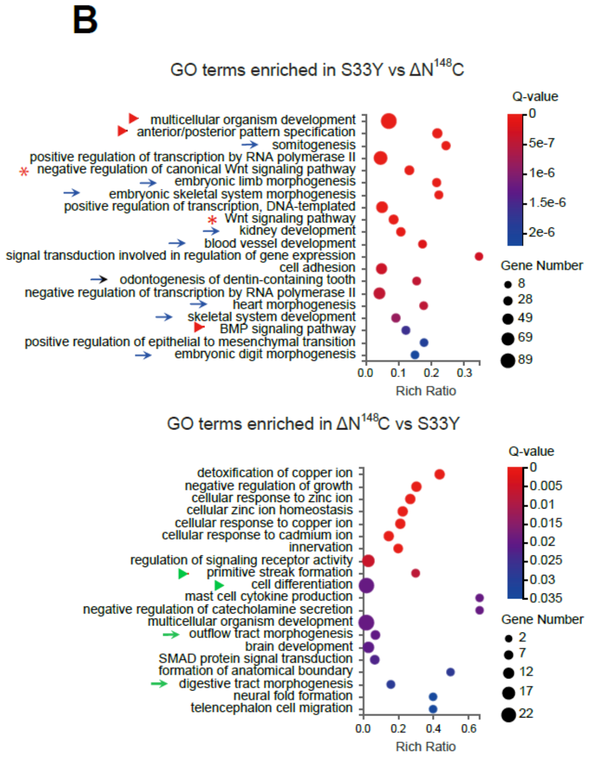

C

Upregulated in S33Y

GO_P Term ID	GO_P Term Desc	Candidate Gene	Q value
GO:0007275	multicellular organism development	CDX1, CDX2, MESP2, DKK1, DKK4, MSX1, MSX2, BMP2, BMP4, TBXT, ROR2, WNT5A etc.	7.24E-31
GO:0009952	anterior/posterior pattern specification	CDX1, CDX2, MSX1, MSX2, TBXT, WNT5A etc.	1.85E-20
GO:0030509	BMP signaling pathway	NKX2-5, ZCCHC12, DLX5, EGR1, MSX1, MSX2, ROR2 , LEF1, TWSG1, BMP2, BMP4, TBXT, CER1	1.46E-06

Upregulated in ΔN148C

GO_P Term ID	GO_P Term Desc	Candidate Gene	Q value
GO:0030154	cell differentiation	FGF8, FOXB1, FOXA2, TCF7L1, etc	0.017
GO:0090009	primitive streak formation	FOXA2, NODAL, GDF3	6.62E-3

Supplementary Fig. S9. S33Y mutant β-catenin activated BMP signaling and mesodermal specification while the ΔN^{148}C induced *NODAL* and endodermal formation (related to Fig. 5)**.**

A. Volcano plot showing differentially expressed genes (DEGs) between S33Y and ΔN^{148}C clones at DE (d1) (FDR ≤ 0.001; FC ≥2). Two vertical dashed lines indicate $\log_2$FC value at –1 and 1. Horizonal dashed line indicates –$\log_{10}$FDR at 3 (FDR=0.001).

B. GO analysis based on the DEGs in **A**. The upper panel shows the GO BPs enriched among the gene upregulated in S33Y clone. Red arrowheads and red asterisks indicate PBs related to BMP and Wnt signaling, respectively. Blue arrows indicate BPs related to the development of mesoderm tissues/organs. The lower panel presents the GO BPs enriched among the gene upregulated in ΔN^{148}C clone. Green arrowheads indicate BPs related to PS formation and endoderm development and green arrows points the development of endoderm tissues.

C. GO BPs marked by the red and green arrowheads in **B,** together with the genes showing upregulation in S33Y (upper) or in ΔN^{148}C (lower) clones.

FDR, false discovery rate; FC, fold change; GO, gene ontology.

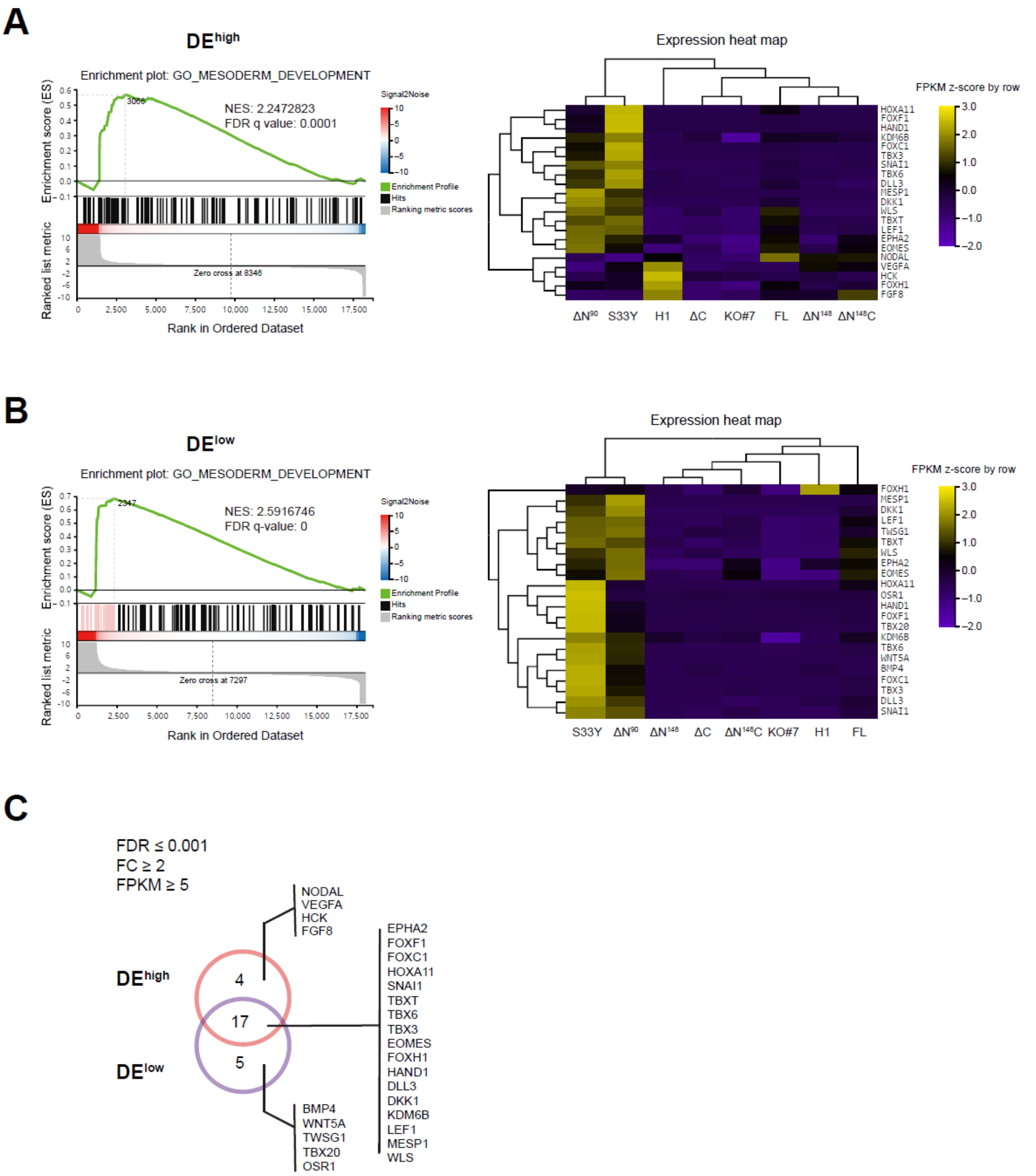

Supplementary Fig. S10. GSEA analysis revealed specific mesodermal induction among DE^{low} but not DE^{high} clones (related to Fig. 5)**.**

A. GSEA analysis of profiling data at DE (d1) in DE^{high} group. The left panel shows the enrichment plot of GO BP for “mesoderm development”, while the right panel shows the expression heatmaps of leading edge set genes (right).

B. GSEA analysis of DE (d1) data in DE^{low} group. The left panel shows the enrichment plot of GO BP for “mesoderm development”, while the right panel shows the expression heatmaps of leading edge set genes (right).

C. Vann diagram showing the overlap between the leading edge genes in **A** and **B**.

NES, normalized enrichment score; FDR, false discovery rate; FC, fold change; GO, gene ontology.

Supplementary Table1. The list of primers used in genome PCR and qRT-PCR.

Gene names	Forward primer Sequences	Reverse primer sequences
Genome PCR primers		
CTNNB1 (F1 & R1)	CTCCTCCTAATGGCTTGGTG	CATAGCAGCTCGTACCCTC
CDH1 (F2 & R2)	CCGGTCTAAGGAAAGTGGGG	GTCTCTACAGTGCTGAGGGC
Dn-F	CAATAGGCCGAAATCGGCAAAATCCC	
Dn-R	CCTCACATTGCCAAAAGACG	
qRT-PCR primers		
CTNNB1 N-ter	ATGCTGCTCATCCCACTAATGTCC	ACCATAACTGCAGCCTTATTAACCAC
CTNNB1 ARM	AGGGGTCCTCTGTGAACTTGCTC	CTTGTAATCTTGTGGCTTGTCCTC
CTNNB1 C-ter	TGGCAACCAAGAAAGCAAGC	TAGCACCTTCAGCACTCTGC
CTNNB1 3'UTR	CAATCAGCTGGCCTGGTTTG	CTCCCACCCTACCAACCAAG
GAPDH	CCAGGGCTGCTTTTAACTCTGGTAAAGTGG	ATTTCCATTGATGACAAGCTTCCCGTTCTC
FOXA2	GTGTACTCCCGGCCCATTATGAACTC	TCCTTATATAGAACGTGGGGTATCTGTGTGG
SOX17	CACGGAATTTGAACAGTATCTGCACTTCG	ACACGTCAGGATAGTTGCAGTAATATACCG
EOMES	ACATTAATGCTGAAGAGTATAGTAAAGACACC	TTTTGGCAACCTAGGCAAAGAAGACAAC
TBXT	TGCTGAACTCCTTGCATAAGTATGAGCC	TTAATTTTAAGAGCTGTGATCTCCTCGTTC
MIXL1	CTCTAGCTCCCAAGGTCAGAATTTTGAAACC	TGCCTCTTCAGAGCTTATCCCGAATTCTC
NODAL	CACTCTGCCATTATCCACATAC	CAAGCAGTACAACGCCTATC
GATA4	GCTCCGTGTCCCAGACGTTCTCAGTCAGTG	TACGCAGTGATTATGTCCCCGTGACTGTCG
GATA6	TGTCACACCACAACTACCACCTTATGGC	TTAGGTTTTCGTTTCCTGGTTTGAATTCCC
CER1	TGGCACCACTTCATGTTCAGAAAAACTCCG	TGAACAGACCCGCATTTCCCAAAGCAAAGG
GSC	TCTCAACCAGCTGCACTGTC	CGTTCTCCGACTCCTCTGAT
CDH1	CCGAGAGAGTTTCCCTACGTATACCCTG	CTTTCAGTGTGGTGATTACGACGTTAGC
TWIST1	CGGCCAGGTACATCGACTTCC	CTCCATCCTCCAGACCGAGAAG
TWIST2	AGCGACGAGATGGACAATAAGATGACC	CGGTCCGGAGGTGGGTGGCG
SNAI1	CAAATACTGCAACAAGGAATACCTCAGC	GTACTTCTTGACATCTGAGTGGGTCT
SNAI2	GCATATTCGGACCCACACATTACC	TTGCACTGGTATTTCTTTACATCAGAATGG
VIM	ACAACCTGGCCGAGGACATC	AGAGACGCATTGTCAACATCCTG

Supplementary Table 2. Summary of RNA-seq samples and data.

Samples	Total clean reads (millions)	Gene mapping		Genome mapping	
		Rate of total mapped genes (%)	Rate of uniquely mapped genes (%)	Rate of total mapped genes (%)	Rate of uniquely mapped genes (%)
H1	45.12	62.3	58.49	91.85	86.63
H1_Dox	44.95	63.71	59.11	91.86	86.65
KO#7_Dox	44.15	64.01	60.33	87.09	82.68
FL_Dox	44.94	67.21	62.26	92.22	86.9
S33Y_Dox	45.42	65.31	61.51	88.3	83.63
ΔN^{90}_Dox	45.01	68.49	63.61	91.2	85.72
ΔN^{148}_Dox	45.06	63.11	59.17	87.68	83.1
ΔN^{148}C_Dox	44.02	63.34	59.65	88.42	83.82
ΔC_Dox	44.71	68.15	63.03	91.94	86.56
H1_DE (d1)	45.28	67.12	62.98	92.36	86.86
H1_Dox_DE (d1)	45.25	65.46	61.33	91.24	85.67
KO#7_DE (d1)	45.49	65.15	61.29	85.42	80.92
FL_ DE (d1)	42.83	66.71	62.01	92.11	86.57
S33Y_DE (d1)	44.72	68.08	64.28	87.64	82.75
ΔN^{90}_DE (d1)	43.87	70.27	65.91	91.57	85.96
ΔN^{148}_DE (d1)	45.25	67.98	63.9	87.9	83.08
ΔN^{148}C_DE (d1)	42.97	68.25	64.2	88.52	83.74
ΔC_DE (d1)	44.82	66.45	61.85	91.06	85.54
